# Taxonomy of three species of the genus *Spinoncaea* (Copepoda, Oncaeidae) in the North Pacific Ocean with focus on morphological variability

**DOI:** 10.3897/zookeys.1043.64438

**Published:** 2021-06-15

**Authors:** Kyuhee Cho, Chailinn Park, Ruth Böttger-Schnack

**Affiliations:** 1 Marine Ecosystem Research Center, Korea Institute of Ocean Science & Technology, Busan, Republic of Korea Marine Ecosystem Research Center, Korea Institute of Ocean Science & Technology Busan Republic of Korea; 2 Global Ocean Research Center, Korea Institute of Ocean Science & Technology, Busan, Republic of Korea Global Ocean Research Center, Korea Institute of Ocean Science & Technology Busan Republic of Korea; 3 Department of Ocean Science, University of Science and Technology, Daejeon, Republic of Korea University of Science and Technology Daejeon Republic of Korea; 4 GEOMAR Helmholtz-Centre for Ocean Research Kiel, Kiel, Germany GEOMAR Helmholtz-Centre for Ocean Research Kiel Kiel Germany

**Keywords:** Molecular, morphological modification, Pacific, taxonomy, zooplankton

## Abstract

Three species of *Spinoncaea* Böttger-Schnack, 2003 are newly recorded in three locations of the equatorial and temperate Pacific Ocean collected by using a net of 60 μm mesh size. For all three species, morphological characters and patterns of ornamentation were analyzed in detail and illustrations of both sexes, also including form variants of the females, are provided. For the first time, information about the variability of various continuous (morphometric) characters are given, such as the spine lengths on the rami of the swimming legs or the proportions of urosomites. The complementary morphological descriptions of the Pacific specimens focus on similarities or modifications of characters as compared to earlier descriptions of these species from the type locality and various other localities. For *S. ivlevi* (Shmeleva, 1966), originally but insufficiently described from the Adriatic Sea, the Pacific material is similar in most aspects to the comprehensive redescription of the species from the Red Sea and from the type locality, except for a difference in the morphometry of the distal endopod segment on the antenna, which is discussed here. For *S. tenuis* Böttger-Schnack, 2003, and *S. humesi* Böttger-Schnack, 2003, the Pacific material mostly coincides with the characteristic features as described in the original account from the Red Sea. For all three species, differences and/or additions in ornamentation details were found in Pacific specimens (e.g., on the intercoxal sclerite of the first swimming leg or on the genital somite of the male) and females with aberrant morphology were detected. Genetic analyses based on 12S srRNA revealed for two species, *S. ivlevi* and *S. humesi*, little or no differences in genetic sequences between Pacific specimens and those recorded from the Mediterranean Sea, thus demonstrating that specimens from both locations are conspecific. For *S. tenuis*, for which no comparable genetic data are available, 12S srRNA amplification was unsuccessful as was the amplification of mitochondrial COI (barcoding) for all three species. The applicability of using COI amplification for barcoding of oncaeid copepods is discussed.

## Introduction

Species of Oncaeidae Giesbrecht, 1893 [1892] are abundant in marine ecosystems of temperate, tropical, and polar regions and in the whole water column (Metz 1995; Böttger-Schnack et al. 2001; Nishibe and Ikeda 2004; Razouls et al. 2005–2021 at http://copepodes.obs-banyuls.fr/en). More than 170 years of taxonomic studies has led to the identification of 113 species ranging from small to large sizes of between 0.17–1.4 mm female body length (Razouls et al. 2005–2021; Walter and Boxshall 2021). All these species are distinguished by morphological analysis using traditional descriptive taxonomy, but they include many sister species, making it difficult to identify them clearly (Böttger-Schnack and Schnack 2013, 2015, 2019).

The genus *Spinoncaea* was established by Böttger-Schnack (2003) to accommodate species of the *ivlevi*-group as deﬁned in a preliminary phylogenetic study of Oncaeidae (Böttger-Schnack and Huys 2001). The typical characteristics of the genus are (1) the modification of caudal rami seta III into a strong spiniform element, (2) the undulate or lobate hyaline frill at the posterior margin of the urosomites and (3) the reduced number of six elements on the maxillule. *Spinoncaea ivlevi* (Shmeleva, 1966), the type species of this genus, was originally described from the Adriatic Sea. Thereafter, Malt (1982) provided a taxonomical report from the Atlantic. In 2003, Böttger-Schnack published a detailed morphological re-analysis of the species, including all the mouthparts, based mainly on copepod material from the Red Sea as compared to specimens from the type locality (Adriatic Sea) and including specimens from various regions in the Indian and Pacific Oceans (cf. Böttger-Schnack 2003: table 3). In the same account, two new species, *S. humesi* Böttger-Schnack, 2003 and *S. tenuis* Böttger-Schnack, 2003 were described, which differed from *S. ivlevi* in the spine count on P2 exopod-3 (*S. humesi*) and/or proportional lengths of the female urosome as well as modifications of caudal setae. Overall, the three described species are very similar in morphology and include some intraspecific variability as observed within as well as between different regions (Böttger-Schnack 2003). Also, females of *S. ivlevi* and *S. tenuis* exhibited two form variants each, which differed mainly in body proportions, especially in the urosomites, and slightly in endopodal spine lengths (Böttger-Schnack 2003). As for *S. ivlevi*, the detection of form variants hampered an unambiguous assignment of either form to the genuine species from the Adriatic Sea (Böttger-Schnack 2003).

Recently, a taxonomic study of the family Oncaeidae has been performed in the NE equatorial Pacific Ocean and one species of *Spinoncaea* identified as *S. ivlevi* was reported (Cho 2011). However, there was some doubt about the identification as the females in Cho’s study showed morphological differences from the genuine *S. ivlevi* female in the proportional length of the female urosome, the length of the second endopod segment of the antenna, and the length ratio of the distal exopod segment to the distal spine on P2–P4.

As a part of a new and ongoing taxonomical study on the oncaeid copepods in the temperate and tropical Pacific, we obtained new copepod material of *Spinoncaea* from the northeastern and northwestern equatorial Pacific as a supplement to the copepod material sampled earlier (Cho 2011) and we also included samples taken in the Korea Strait. All three *Spinoncaea* species were found and examined in great detail. The present paper provides redescriptions of the morphological characters of the three species of *Spinoncaea* in these locations of the Pacific Ocean. Particular attention was paid to the variability of continuous morphological characters, such as e.g., the spine lengths on the rami of the swimming legs, as well as the occurrence of form variants among the females. In comparison to the earlier descriptions by Böttger-Schnack (2003) morphological differences and additions will be provided and the importance of information on the morphological variability within these species will be discussed. In addition, we performed genetic analysis to test the hypothesis that species with morphological variation will show genetic differences. To compare the sequences of *Spinoncaea* species with those obtained by Böttger-Schnack and Machida (2011) from the Mediterranean Sea, specimens from the Pacific were analyzed by the genetic regions of the mitochondrial cytochrome c oxidase subunit 1 (mtCOI) and 12S small ribosomal RNA (12S srRNA).

## Material and methods

**Figure 1.**
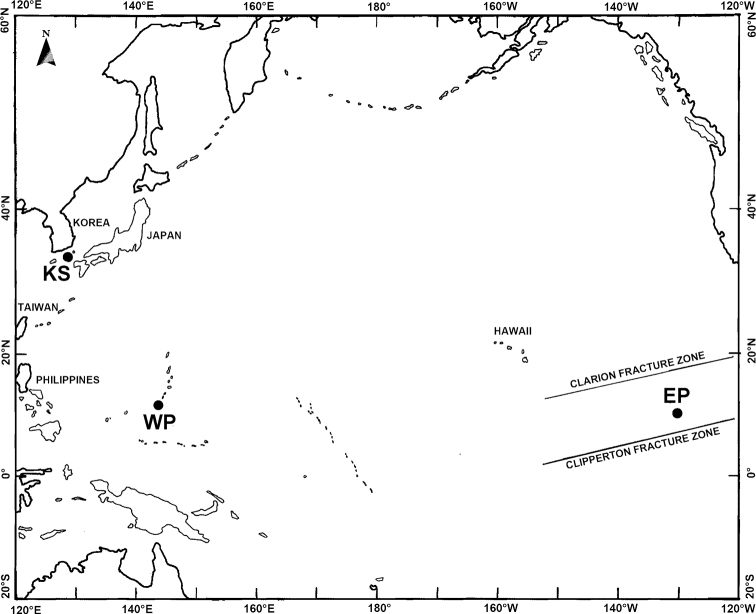
Location of the sampling stations in the northeastern equatorial Paciﬁc (EP), the northwestern equatorial Pacific (WP), and the Korea Strait (KS).

The copepod material was collected in three different regions and years in the Pacific Ocean, in the tropical northeastern (EP-1; 21 August 2009, EP-2; 19 March 2019) and northwestern (WP-1; 27 March 2016, WP-2; 4 April 2016) Pacific, and in the Korea Strait (KS; 7 October 2008) (Fig. 1). A conical net (60 cm mouth diameter, 60 µm mesh size) was used to sample different integrated vertical depth layers in the epipelagic zone between 0–100 m and 0–200 m. A station list with geographic positions, dates and depth layers sampled is given in Table 1. Each net sample was preserved in 99.9% ethyl alcohol immediately after collection on board. In the laboratory, oncaeids were sorted out from the preserved zooplankton samples under a stereomicroscope (Semi 2000-C; Carl Zeiss, Germany). Specimens were dissected with tungsten needles, mounted in lactophenol: glycerin (1:5), and sealed with transparent nail-varnish. Some specimens were mounted in fluoromount-G (SouthernBiotech, Birmingham, USA) on H-S slides (Double slide plate, BSDS-011R; Biosolution, Republic of Korea) (cf. Shirayama et al. 1993). For the purpose of morphometries and illustrations a differential interference contrast light microscope (DM2500; Leica, Wetzlar, Germany or BX51; Olympus, Tokyo, Japan) with a drawing tube was used. To prepare specimens for scanning electron microscope analysis (S-4300; Hitachi, Tokyo, Japan), specimens were fixed with 2% Glutaraldehyde and 2% OsO_4_, dehydrated with graded ethanol, substituted with t-BuOH, dried by freeze dryer (ES-2030; Hitachi, Tokyo, Japan), mounted on stubs using copper tape, coated with platinum using an ion sputter (E-1045; Hitachi, Tokyo, Japan), and then photographed. Some specimens were deposited in the collection of National Institute of Biological Resources (**NIBR**), Incheon, Korea and the accession numbers are written in parentheses next to the specimens.

**Table 1.** Sample locations for species of *Spinoncaea* in the equatorial and temperate Pacific Ocean.

Region	Station	Date	Geographical position	Sampling depth (m)
Northeastern Pacific	EP-1	21 August 2009	10°23'N, 131°20'W	100
	EP-2	19 March 2019	9°52'1.38"N, 131°45'38.28"W	200
Northwestern Pacific	WP-1	27 March 2016	13°23'46.44"N, 143°55'0.6"E	150
	WP-2	4 April 2016	13°20'3.42"N, 144°20'2.7"E	150
Korea Strait	KS	7 October 2008	33°44'50.50"N, 128°15'39.02"E	110

The morphological terminology used in the text and figures was adopted from Huys et al. (1996). Abbreviations:

**Al** antennule;

**A2** antenna;

**ae** aesthetasc;

**P1–P6** first to sixth thoracopod;

**exp** exopod;

**enp** endopod;

**exp** (enp)-1 (2, 3) to denote the proximal (middle, distal) segment of a three-segmented ramus.

Body sizes of individuals were measured laterally from the anterior margin of the prosome to the posterior margin of the caudal rami, not considering the various degrees of telescoping of somites. The length to width ratio of the caudal rami, the anal somite, and the genital (double-)segment was measured in dorsal view. The variability of individual spine lengths on the exo- and endopod segments of the swimming legs was examined by calculating (1) on the exopods of P2–P4 (1a) the length of the distal exopod segment in relation to the length of the distal spine; (1b) the length of the outer spine on the proximal exopod segment in relation the outer spine on the middle exopod segment; (1c) the length ratio of the outer spine on the proximal exopod segment compared to the length of the outer spines on the distal exopod segment; (2) on the endopods of P2–P4, the length of the outer subdistal and/or outer distal spine on the distal segment in relation to the length of the distal spine. If possible, both the left and right sides of the swimming legs were measured for each specimen. Scale bars in the figures are indicated in micrometers (μm).

Total genomic DNA was extracted from presorted single individuals with DNeasy Blood & Tissue Kit (Qiagen, Hilden, Germany) following the protocol of Cornils (2014). PCR amplifications were performed targeting mitochondrial COI and the 12S small ribosomal RNA genes. Two sets of primers, mtCOI primers [LCO1490, HCO2198 (Folmer et al. 1994)] and 12S srRNA primers [L13337-12S (Machida et al. 2002), H13842-12S (Machida et al. 2004)] were used for gene amplification. PCR reactions were carried out in 20 μl containing 5 μl of template, 0.2 μl of 2.5 unit Z-Taq (Takara, Kusatsu, Japan), 1 μl of each primer (5 μM), 2 μl of dNTP (2.5 mM each), 2 μl of 10X buffer, 0.6 μl of DMSO (99%), and 8.2 μl of sterile distilled water. The cycling profile was denaturation at 94 °C for 5 sec, annealing at 48 °C for 5 sec, and extension at 72 °C for 10 sec, for 40 cycles in the C1000 Touch Thermal Cycler (Bio-Rad, California, USA). PCR products were stained with Loanding STAR (Dyne Bio, Seongnam, Republic of Korea) and electrophoresed in 1.5% agarose gel. Positive PCR products were purified with AccuPrep PCR/Gel Purification Kit (Bioneer, Daejeon, Republic of Korea) and sent to Macrogen (Seoul, Republic of Korea) for sequencing.

DNA sequences were compared against known species from the NCBI GenBank nucleotide database using BLASTn. All sequences were edited using BioEdit 7.0.5.3 software (Hall 1999). Edited sequences were aligned by ClustalW using MEGA 7 software (Kumar et al. 2016). The phylogenetic tree was constructed by Maximum-likelihood with Kimura two-parameter distance in MEGA 7 software.

## Results

### Systematics

**Order Cyclopoida Burmeister, 1834**

#### Family Oncaeidae Giesbrecht, 1893 [1892]

**Genus *Spinoncaea* Böttger-Schnack, 2003**

The morphology of the three *Spinoncaea* species from the Pacific agrees in general with the (re)-description of these species from the Red Sea (Böttger-Schnack 2003), but a number of additions, modifications and/or supplements were found, which are specified in the following for each species and form variant. As the variability of morphometric data was studied for the Pacific specimens only (Tables 3, 4), the respective individual values of Red Sea specimens are mentioned only when outside the range of values from the Pacific.

##### Spinoncaea ivlevi

Taxon classificationAnimaliaCyclopoidaOncaeidae

(Shmeleva, 1966)

E63B3C5F-7FA2-574C-A978-3806118B21F6

**Figure 2.**
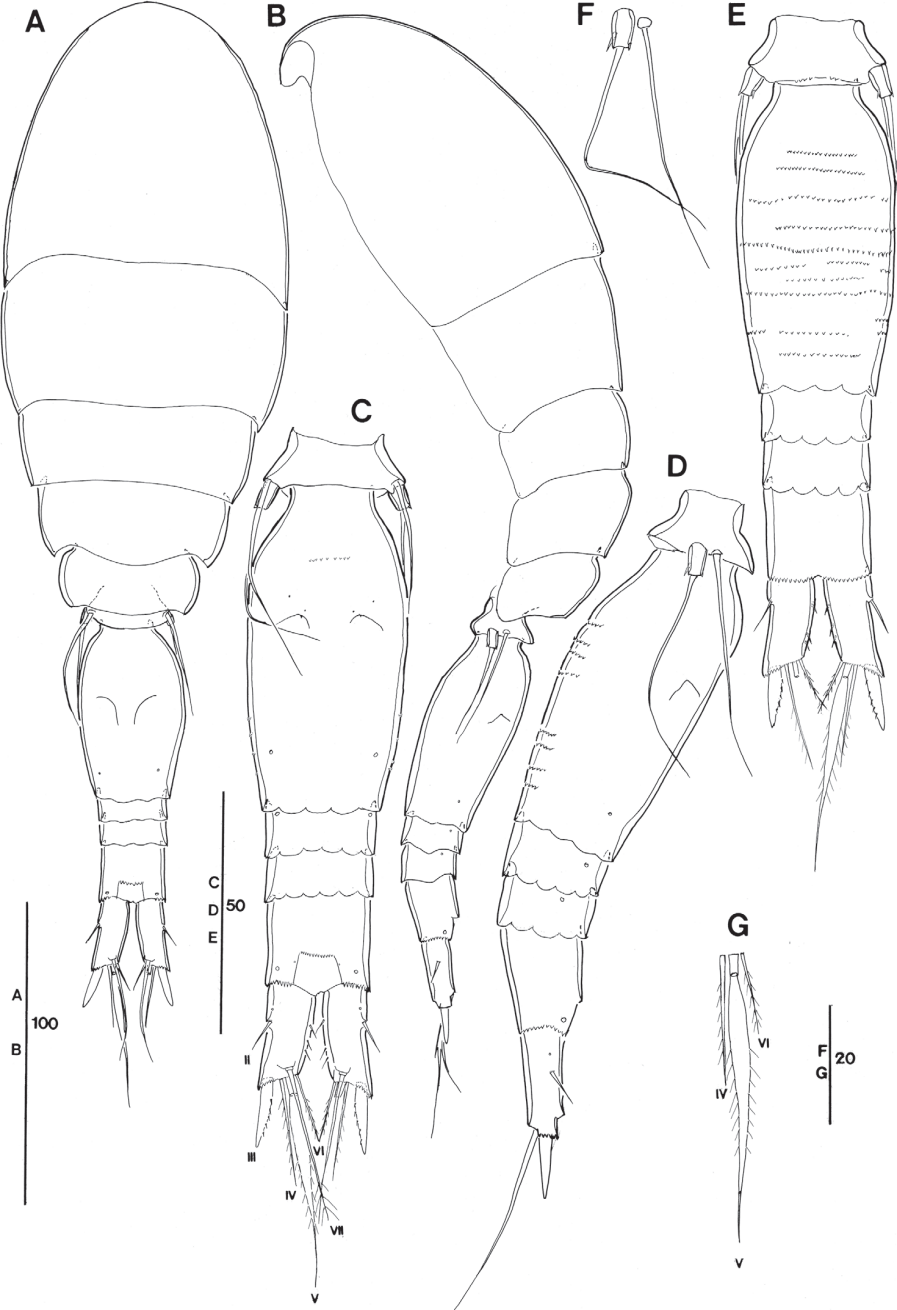
*Spinoncaea ivlevi* (Shmeleva, 1966), female, robust form (northwestern equatorial Pacific) **A** habitus, dorsal (caudal seta V on right side missing) **B** habitus, lateral **C** urosome, dorsal, setae on caudal rami are numbered using Roman numerals (seta V on right side missing) **D** urosome, lateral (seta V on right side missing) **E** urosome, ventral **F** leg 5, lateral **G** caudal setae IV–VI shown separately. Scale bars in μm.

**Figure 3.**
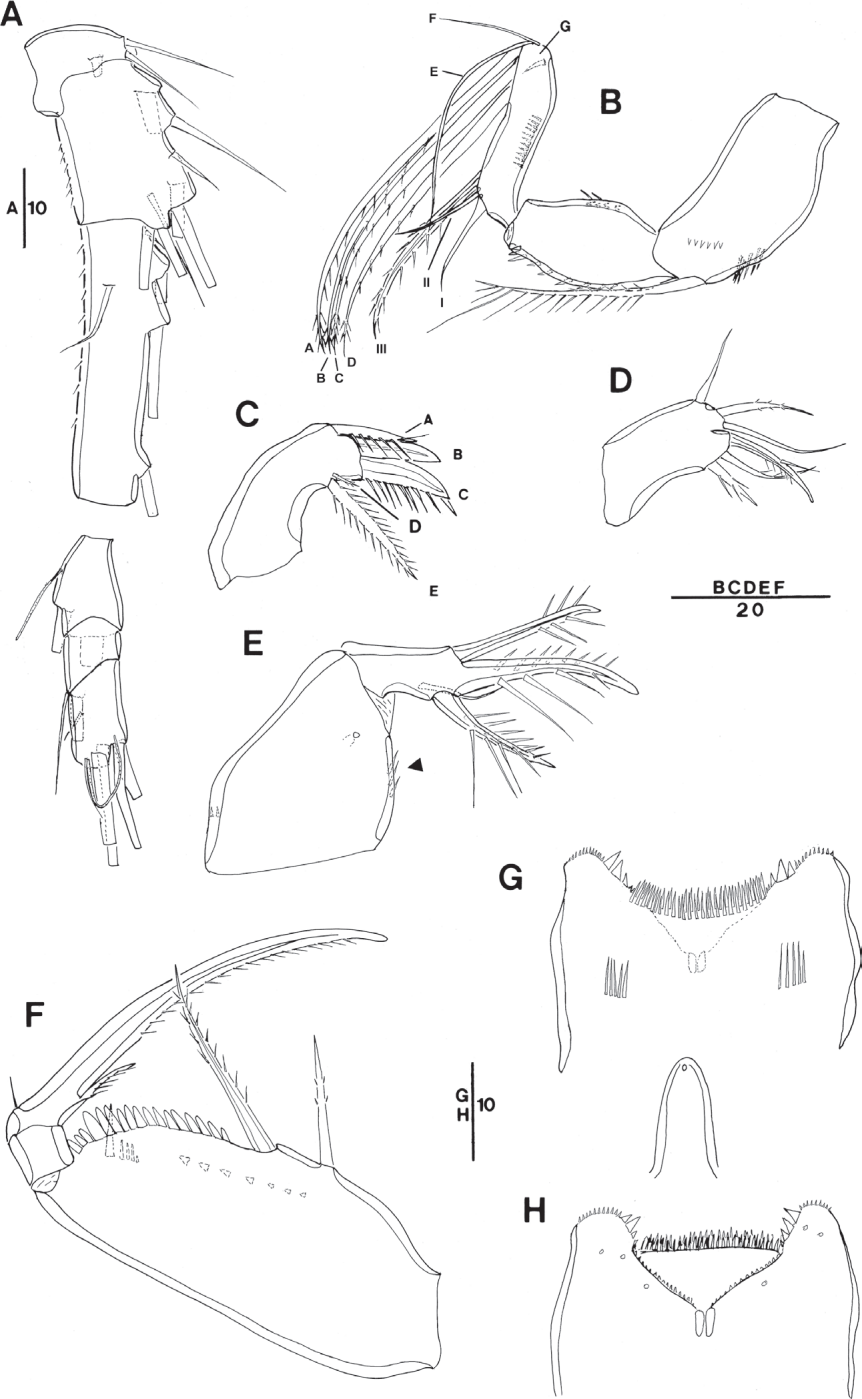
*Spinoncaea ivlevi* (Shmeleva, 1966), female, robust form (northwestern equatorial Pacific) **A** antennule (separated between segments 3 and 4) **B** antenna, distal elements on distal endopod segment numbered using capital letters, lateral elements indicated by Roman numerals **C** mandible, individual elements indicated by capital letters **D** maxillule **E** maxilla, arrow indicating spinules on syncoxa, **F** maxilliped, posterior, syncoxa missing **G** labrum, anterior **H** labrum, posterior. Scale bars in μm.

**Figure 4.**
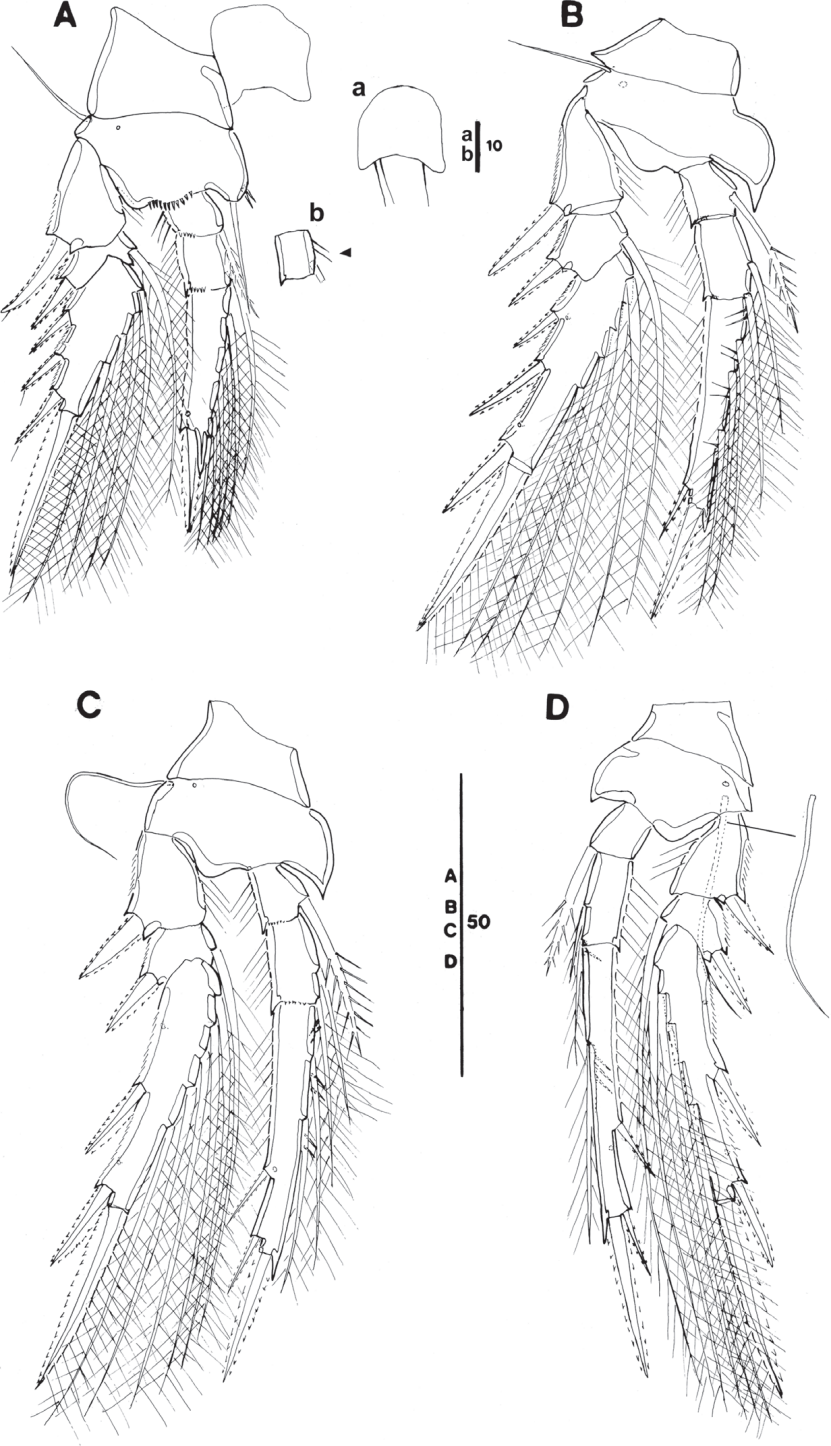
*Spinoncaea ivlevi* (Shmeleva, 1966), female, robust form (northwestern equatorial Pacific) **A**P1, anterior [a: ornamentation on intercoxal sclerite of another specimen, b: second endopod segment shown separately, strong setules on inner margin arrowed] **B**P2, posterior **C**P3, anterior **D**P4, anterior, seta on basis figured separately. Scale bars in μm.

**Figure 5.**
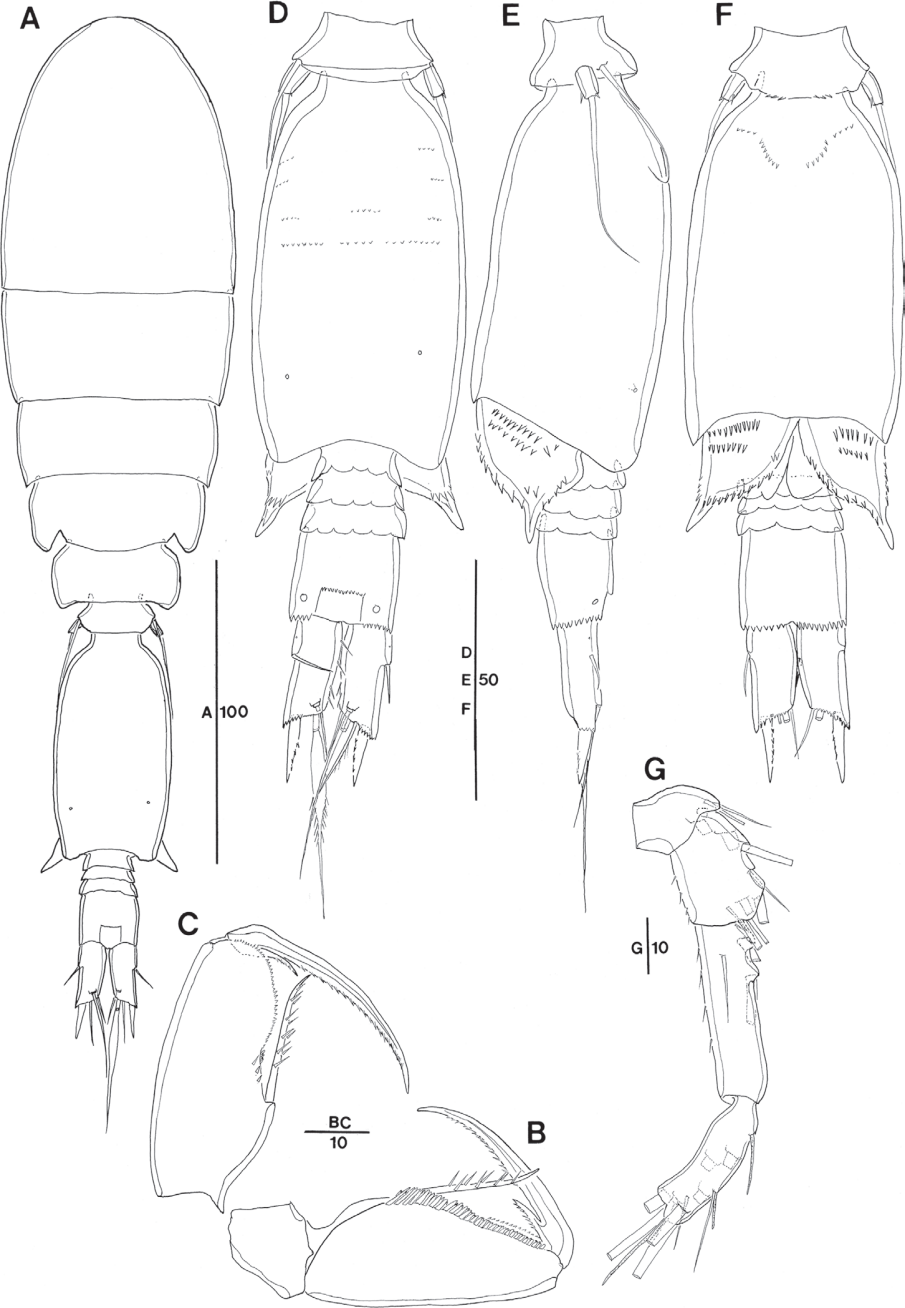
*Spinoncaea ivlevi* (Shmeleva, 1966), male (northwestern equatorial Pacific) **A** habitus, dorsal (caudal seta V on left side missing) **B** maxilliped, posterior **C** maxilliped, anterior, syncoxa missing **D** urosome, dorsal (caudal seta IV on left side and seta V on right side missing, seta VII on left side omitted) **E** urosome, lateral **F** urosome, ventral (caudal seta IV on left side and seta V on right side missing, seta IV on right side and seta V on left side omitted) **G** antennule. Scale bars in μm.

**Figure 6.**
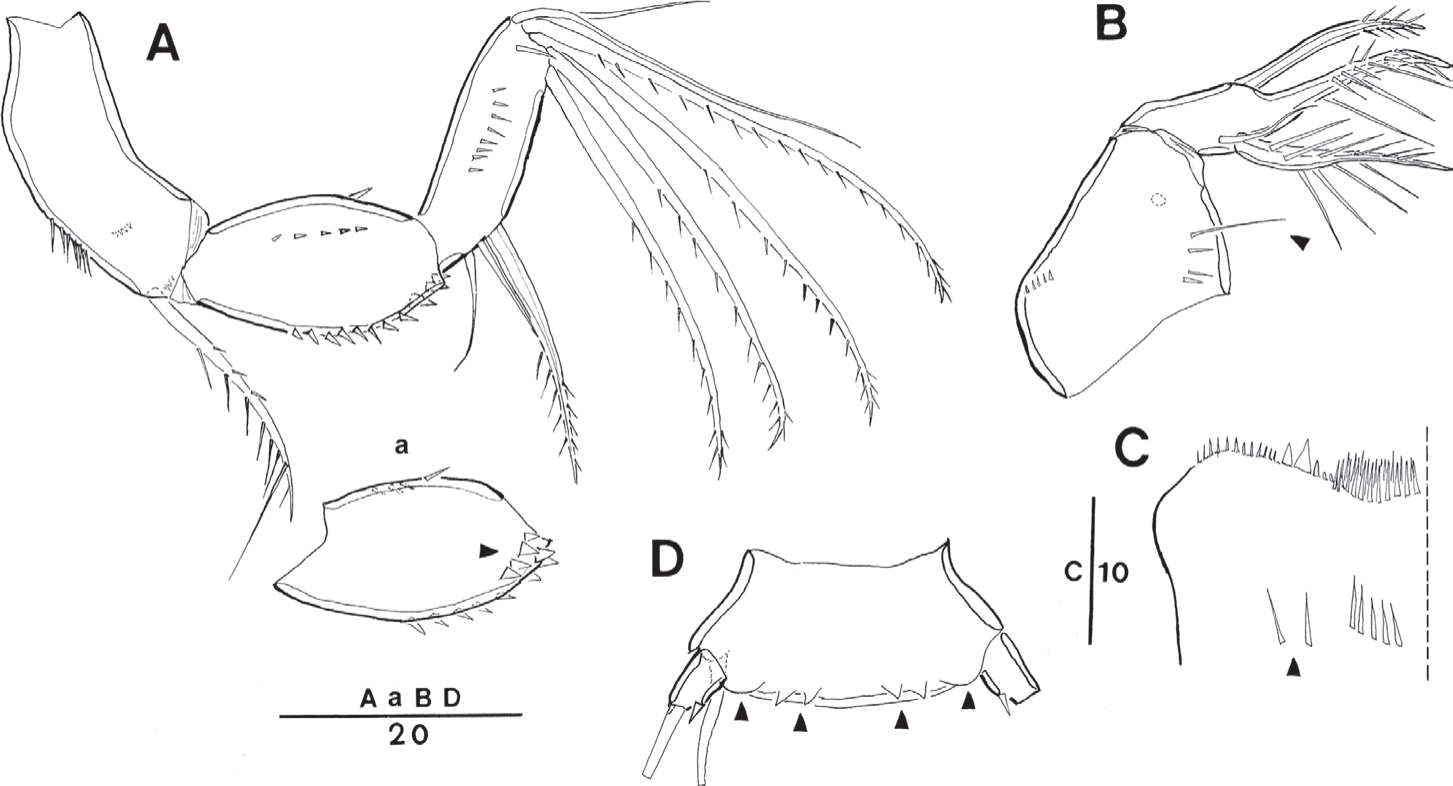
*Spinoncaea ivlevi* (Shmeleva, 1966), female, robust form (Korea Strait) **A** antenna [a: first endopod segment of right antenna, additional broad spinules arrowed] **B** maxilla, long setule arrowed **C** labrum, anterior, additional setules arrowed **D**P5-bearing somite, ventral, midventral spinous processes and weakly pronounced ventrolateral lobes arrowed. Scale bars in μm.

**Figure 7.**
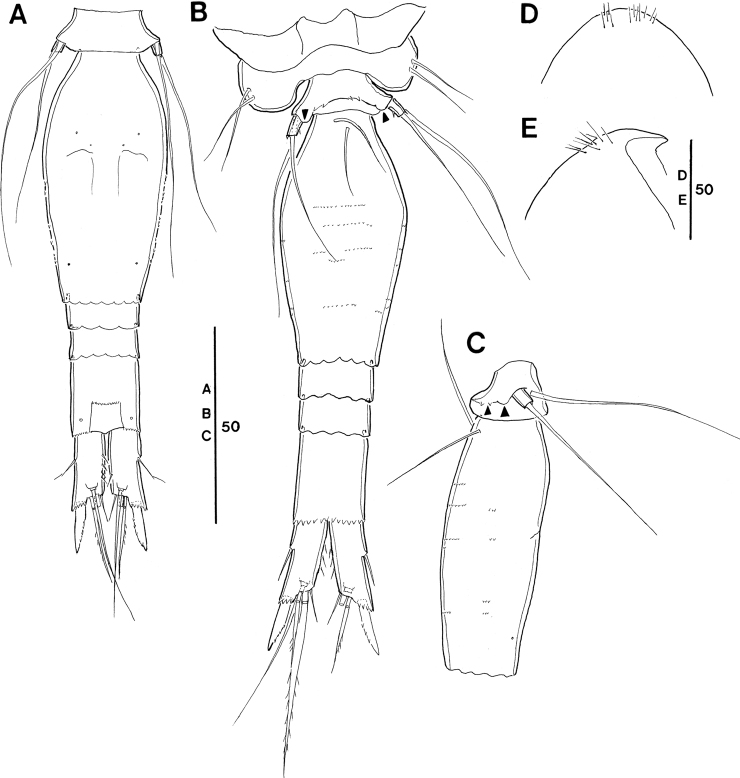
*Spinoncaea ivlevi* (Shmeleva, 1966), female, elongate form, general (northwestern equatorial Pacific) **A** urosome, dorsal, caudal seta V on both sides missing; elongate form, aberrant (northeastern equatorial Pacific) **B**P4-bearing somite and urosome, ventral, showing ornamentation on P4 and P5-bearing somites and genital double-somite, ventrolateral lobes arrowed, caudal seta V on left side missing **C**P5-bearing somite and genital double-somite, lateral, showing 2 long setules on ventral side of double-somite, midventral spinous process and ventrolateral lobe arrowed. *Spinoncaea ivlevi* (Shmeleva, 1966), female, robust form, variation, (northwestern equatorial Pacific) **D** setular patch on tip of cephalosome, dorsal **E** setular patch on cephalosome, lateral. Scale bars in μm.

Figs 2 , 3 , 4 , 5 , 6 , 7 , 16

Oncaea ivlevi Shmeleva, 1966: 932–933, figs 1.1–1.11 (Adriatic).
Oncaea ivlevi : Shmeleva 1969: 5–8, 27, figs 3a–i, 4a–h (Adriatic, tropical Atlantic).
Oncaea ivlevi : Malt 1982: 186–187, 193, figs 3a–k, 4a–d (temperate Atlantic).
Spinoncaea ivlevi : Böttger-Schnack 2003: 193–207, figs 2–7 (Red Sea, Mediterranean Sea, Indian and Pacific oceans).

###### Material examined.

**1. Robust form.** (1) Northwestern Pacific (a) 13°23'46.44"N, 143°55'0.60"E (WP-1), 27 March 2016: Five females and four males dissected on several slides, respectively. Four dissected females (NIBRIV0000882743–882746) and four dissected males (NIBRIV0000882747–882750) were deposited in the NIBR; (b) 13°20'3.42"N, 144°20'2.7"E (WP-2), 4 April 2016: Six females dissected on several slides, respectively. Four dissected females (NIBRIV0000882751–882754), one undissected female (NIBRIV0000882755) and one undissected male (NIBRIV0000882756) mounted on H-S slide, respectively, and five undissected females and three undissected males in alcohol vial (NIBRIV0000882757) were deposited in the NIBR. (2) Northeastern Pacific, 10°30'N, 131°20'W (EP-1), 21 August 2009: Six females (NIBRIV0000882758–882763) and four males (NIBRIV0000882764–882767) dissected on several slides, respectively. All dissected specimens, one undissected female (NIBRIV0000882768) and one undissected male (NIBRIV0000882769) on respective H-S slide, and five undissected females and two undissected males in alcohol vial (NIBRIV0000882770) were deposited in the NIBR. (3) Korea Strait, 33°44'50.50"N, 128°15'39.02"E (KS), 7 October 2008: Three females (NIBRIV0000882771–882773) and one male dissected (NIBRIV0000882774) on H-S slide, respectively. All dissected specimens and two undissected females and two undissected males in alcohol vial (NIBRIV0000882775) were deposited in the NIBR.

**2. Elongate form.** (1) Northwestern Pacific, 13°23'46.44"N, 143°55'0.60"E (WP-1), 27 March 2016: One female (NIBRIV0000882776) dissected on two slides. This specimen was deposited in NIBR. (2) Northeastern Pacific, 10°30'N, 131°20'W (EP-1), 21 August 2009: Three females (NIBRIV0000882777–882779) dissected on one slide or three slides, respectively. Two females (aberrant) (NIBRIV0000882780) dissected on H-S slide. The morphometric data provided in Tables 3 and 4 included only four specimens (three normal females and one aberrant female). All dissected specimens except for one specimen of aberrant female and one undissected aberrant female (in alcohol, NIBRIV0000882781) were deposited in the NIBR.

###### Description.

**Female (robust form, Figs 2–4, 6, 7D, E, 16A–D, Tables 3, 4).** Body length (in lateral view, telescoping of somites not considered) range 318–373 µm in Pacific specimens (Table 3), showing a wider size range than in the Red Sea (330–340 µm, Böttger-Schnack 2003: 193).

Prosome 1.9 × length of urosome, excluding caudal rami, 1.6 × urosome length, including caudal rami (Fig. 2B), calculated by not correcting for the telescoping of somites. Variation of prosome to urosome length (including CR) ratio 1.5–1.7 in Pacific specimens (Table 3). The respective values provided for Red Sea specimens are not comparable as they were based on length data corrected for the telescoping of somites. When calculating the body proportions of the female from Böttger-Schnack’s fig. 2A by not correcting for the telescoping of somites, the respective ratio of prosome to urosome length (incl. CR) would account to 1.5, which is within the range of values for Pacific specimens.

P5-bearing somite with paired row of midventral spinous processes (Fig. 2E), variable in number, generally two or three processes, difference per body side may appear as in Fig. 2E: four (right) and two (left). No such variation was mentioned for the Red Sea specimens.

Posterior margin of genital double-somite and postgenital somites with undulate hyaline frill (Fig. 2C, E), as typical for *Spinoncaea* species, shown in detail in Fig. 16D.

Genital double-somite (Figs 2C, D, E, 16D) 2.0 × as long as maximum width in specimen figured (measured in dorsal aspect) and ~ 1.5 × as long as postgenital somites combined; variation in length to width ratio 1.6–2.0 in Pacific specimens (Table 3), surface ornamentation and pore pattern as figured (Figs 2E, 16D).

Anal somite approximately as wide as long, with insignificant variation in length to width ratio (Table 3), ornamentation as figured (Fig. 2C, E).

Caudal ramus (Fig. 2A, C, G) with length to width ratio 1.9–2.2 measured along inner margin and 2.4–2.9 measured along outer margin (Table 3). Caudal seta II with a single long spinule (as in male, e.g., Fig. 16E), which is difficult to discern, and which was not reported for Red Sea specimens, and seta IV with ornamentation being unipinnate, while it is bipinnate in Red Sea specimens. Variation in length ratios among setae II, III, and IV as given in Table 3, denoting a smaller ratio for seta III:II (1.3–1.9) than in the Red Sea (2.2; Böttger-Schnack 2003: fig. 2F).

Antennule 6-segmented (Fig. 3A) with armature formula: 1-[3], 2-[8], 3-[5], 4-[2+ae], 5-[2 (ae not discernible)], 6-[5+(1+ae)], typical for *Spinoncaea* species.

Antenna 3-segmented, armature as for Red Sea specimens, including the absence of seta IV on the lateral armature of the distal endopod segment (Fig. 3B, setae I–III indicated). Distal endopod segment reflexed (Fig. 3B), 3.0–3.9 × longer than wide (Table 3), somewhat longer than reported for Red Sea specimens (ca 3:1; discussed under “Remarks”). Ornamentation of elements differing slightly from Red Sea specimens in (1) the coxobasis with a long seta at inner distal corner is ornamented with long spinules unilaterally along entire length, including a single very long spinule at distal part, but only a short row of small spinules at anterior half (Fig. 3B), while in specimens from the Red Sea this seta is ornamented with strong spinules bilaterally and lacking a single long spinule (Böttger-Schnack 2003: fig. 3A), and on (2) the proximal endopod segment is lacking single strong spine on expanded outer margin in specimen figured (Fig. 3B), but is present in specimen from Korea Strait (Fig. 6A), as specified for Red Sea specimens.

Labrum (Figs 3G, H, 16A) showing variable ornamentation on anterior surface, paired row of long setules in specimen figured (Fig. 3G, indicated by white arrow in Fig. 16A) as specified for Red Sea specimens, additional row of setules indicated in specimen from Korea Strait (Fig. 6C).

Mandible (Fig. 3C) gnathobase with five elements, with dorsal element D shortest and inserting near base of seta E, as typical for *S. ivlevi* (cf. Böttger-Schnack 2003: 191) difficult to discern in some specimens from the Pacific.

Maxillule (Figs 3D, 16B) with six elements [innermost element on outer lobe absent, as typical for *Spinoncaea* species]; ornamentation of middle and innermost element on inner lobe as well as of element next to innermost on outer lobe (Fig. 16B) slightly modified as compared to Red Sea specimens.

Maxilla (Fig. 3E) with additional ornamentation on syncoxa showing rows of short spinules along outer margin and long spinules along inner margin (arrowed in Fig. 3E), not reported earlier for Red Sea specimens.

Maxilliped (Fig. 3F, syncoxa missing) with basis ornamented with fringe of short spatulated spinules between distal seta and articulation with endopod, as illustrated for Red Sea specimens (Böttger-Schnack 2003: fig. 3G, but erroneously described as “…between proximal seta and articulation with endopod;..” in text on p 200).

Swimming legs 1–4 (Fig. 4A–D) with armature formula shown in Table 2. Intercoxal sclerite of P1 ornamented with paired long, fine setules (Figs 4a, 16C), which were not discernible in some specimens. Outer seta on basis of P1 slightly shorter than in Red Sea specimens and naked. Anterior surrounding of bases of spines on exopodal and endopodal segments (= small spinules) not discerned in Pacific specimens.

**Table 2.** Swimming legs armature formula. Roman numerals indicate spines, Arabic numerals represent setae. Differences in spine count are marked in bold. (a) *S. ivlevi* and *S. tenuis*, (b) *S. humesi*.

Leg	Coxa	Basis	Exopod	Endopod
P1	0–0	1–I	I-0; I-1; III,I,4	0–1; 0–1; 0,I,5
P2	0–0	1–0	I-0; I-1; **III^(^**^a)^/**II**^(b)^,I,5	0–1; 0–2; 0,II,3
P3	0–0	1–0	I-0; I-1; II,I,5	0–1; 0–2; I,II,2
P4	0–0	1–0	I-0; I-1; II,I,5	0–1; 0–2; I,II,1

Exopods with general characteristics as for Red Sea specimens, including a reduced length of spine on middle segment (= exp-2) of P2 and P3 (Fig. 4B, C) and of proximal spine on distal segment (= exp-3) of P2 (Fig. 4B); variability of proportional spine lengths, however, indicates that extent of size reduction of spine on exp-2 differs between legs: most obvious on P2, less obvious on P3 and insignificant on P4 (Table 4). Distal spine on P1 slightly longer, on P2–P4 shorter than distal exopod segment, variability of respective length ratios (Table 4) indicating that the respective size difference is less obvious in P2 as compared to P3 and P4.

Endopods with length ranges of outer subdistal spine and outer distal spine relative to distal spine given in Table 4 generally similar to Red Sea specimens (Böttger-Schnack 2003: fig. 4A–D).

P5 (Fig. 2C, D, F) with length to width ratio of exopod segment 1.6, as for Red Sea specimens.

P6 (Fig. 2C) represented by operculum closing off each genital aperture; possibly armed with a short spinule, which is difficult to discern in Pacific specimens.

**Female (elongate form, Fig. 7A**–**C, Tables 3, 4).** Body length range 305–345 μm, based on five specimens from tropical northeastern and northwestern Pacific, not significantly different from robust form (Table 3).

**Table 3.** Variation in morphometric traits for three species of *Spinoncaea* (both sexes, including form variants of female *S. ivlevi*) from three locations in the Pacific Ocean (WP = western equatorial Pacific; EP = eastern equatorial Pacific; KS = Korea Strait; n = number of specimens examined) Abbreviations: A2 = antenna; AS = anal somite; CR = caudal ramus, for numbering of setae see Fig. 2C; DES = distal endopod segment; DS = distal spine; G(D)S = genital (double-)somite; in = inner margin; out = outer margin; L = length; PGS = postgenital somites; Pro = prosome; Uro = urosome; W = width.

		*S. ivlevi*								*S. tenuis*				*S. humesi*		
		female					male			female		male		female	male	
		WP		EP		KS	WP	EP	KS	EP	KS	EP	KS	EP	WP	KS
		robust	elongate	robust	elongate	robust										
n		11	1	6	4	3	4	4	1	8	1	3	1	3	2	1
Total length (μm)		327–360	305	318–373	330–345	343–344	306–329	307–331	298	320–355	329	310–325	292	344–348	285/295	292
Pro:Uro including CR		1.5–1.7	1.4	1.5–1.7	1.3	1.5–1.6	1.3–1.5	1.5–1.6	1.4	1.5–1.7	1.3	1.5	1.4	1.3–1.4	1.1/1.2	1.3
AS L:W		1.0–1.1	1.3	1.0–1.1	1.2–1.4	1.0–1.1	0.9–1.1	0.9–1.0	0.9	1.1–1.3	1.2	1.0	1.2	1.2–1.3	1.2	1.2
CR	L:W (in)	1.9–2.2	2.0	1.9–2.2	2.0–2.1	2.1	1.8–1.9	1.7–2.0	1.8	1.8–2.5	2.5	1.9–2.2	2.4	2.3–2.5	2.1/2.2	2.5
	L:W (out)	2.4–2.9	2.5	2.4–2.6	2.6–2.8	2.4	2.3–2.7	2.3–2.5	2.4	2.3–2.9	3.0	2.2–2.6	2.8	2.8–3.1	2.6/2.9	3.2
	Setae III:II	1.5–1.9	2.0	1.3–1.7	1.6–2.0	1.3–1.6	1.6–1.9	1.2–1.9	1.4	1.0–1.5	1.1	1.2–1.5	1.2	1.6–1.7	1.6/1.8	1.3
	Setae IV:III	1.2–1.8	1.7	1.5–1.7	1.2–1.3	1.8–1.9	1.3–1.5	1.2–2.0	1.8	1.4–2.3	2.2	1.7–1.9	2.0	1.2–1.6	1.4/1.5	1.6
G(D)S (dorsal view)	L:W	1.8–2.0	2.0	1.6–1.9	1.9–2.2	1.8	1.8–1.9	1.8–1.9	1.8	1.8–2.3	2.2	1.8–1.9	2.0	1.9–2.0	1.7/1.9	2.1
G(D)S L : PGS L		1.4–1.5	1.7	1.7	1.4–1.5	1.6	2.3–2.5	2.0	2.1	1.5–2.1	1.9	2.6–2.7	2.4	1.4–1.5	2.1/2.2	2.3
G(D)S Max W: Min W (posterior portion)		1.2–1.5	1.5	1.4–1.5	1.4–1.6	1.3–1.5	-	-	-	1.5–2.1	1.8	-	-	1.7–2.0	-	-
A2DES L:W		3.4–3.9	2.2	3.0–3.8	3.4–3.9	3.2–3.9	3.2–3.9	3.6–3.8	-	3.3–4.1	4.1	3.6–4.0	3.8	3.7–4.3	3.8–4.1	3.5

Prosome 1.3–1.4 × length of the urosome (incl. CR), smaller than in the robust form (1.5–1.7, Table 3).

Genital double-somite with shape slightly different from robust form, degree of tapering being stronger (Fig. 7A) than in robust form (Fig. 2C). Length to width ratio of the genital double-somite (1.9–2.2) slightly larger than in robust form (1.6–2.0), but values overlap (Table 3).

Anal somite with length to width ratio larger in elongate form (1.2–1.4) than in robust form (1.0–1.1) (Table 3); longer than CR (measured along outer margin) while in the robust form the anal somite is shorter than the CR (cf. Fig. 2A, C, E).

Caudal ramus with ranges in length to width ratio overlapping between the two female form variants (Table 3).

Antennule (not figured) 6-segmented. Armature formula as for *S. ivlevi* robust form.

Antenna (not figured) 3-segmented, armature as for *S. ivlevi* robust form. Distal endopod segment with variation of length to width (Table 3).

Mandible, maxillule, maxilliped (not figured) similar to those of the robust form.

Swimming legs variable in proportional lengths of endopodal and exopodal spines on P2–P4 as given in Table 4, showing similar ranges of variation among both forms of the species (cf. Table 4).

**Table 4.** Variation in proportional spine lengths on P2–P4 for three species of *Spinoncaea* (both sexes, including form variants of female *S. ivlevi*) from three locations of the Pacific Ocean (WP = western equatorial Pacific; EP = eastern equatorial Pacific; KS = Korea Strait, n = number of specimens measured) Abbreviations: DS = distal spine; L = length; MS = middle spine; ODS = outer distal spine; OSDS = outer subdistal spine; PS = proximal spine; SP = spine; W = width.

		*S. ivlevi*								*S. tenuis*				*S. humesi*		
		female					male			female		male		female	male	
		WP		EP		KS	WP	EP	KS	EP	KS ^*^	EP	KS ^*^	EP	WP	KS ^*^
		robust	elongate^*^	robust	elongate	robust										
n		11	1	6	4	3	4	4	1	8	1	3	1	3	2	1
P2 L ratio exp-3:DS		1.03–1.38	1.13	1.19–1.32	1.03–1.20	1.1–1.29	1.07–1.28	1.20–1.30	1.19	0.92–1.09	0.98	1.02–1.07	1.0/1.03	1.04–1.12	1.04–1.23	1.13/1.16
P3 L ratio exp-3:DS		1.21–1.52	1.21	1.09–1.38	1.06–1.19	1.22–1.43	1.17–1.57	1.23–1.32	1.28	0.95–1.07	0.98	1.01–1.12	1.05/1.06	1.14–1.21	1.13–1.19	1.19/1.22
P4 L ratio exp-3:DS		1.26–1.49	1.26/1.60	1.31–1.41	1.20–1.30	1.26–1.43	1.23–1.52	1.33–1.49	damaged	0.96–1.13	0.97	1.00–1.04	0.99	1.11–1.20	1.06–1.28	1.28/1.48
L ratio spines on P2exp	SPexp-1:SPexp-2	1.24–1.57	1.46	1.33–1.63	1.32–1.43	1.43–1.74	1.44–1.77	1.31–1.55	1.39	1.29–1.47	1.27	1.36–1.52	1.21/1.33	1.31–1.42	1.25–1.32	1.25/1.26
	SPexp-1:PSexp-3	1.36–1.76	1.46	1.36–1.79	1.46–1.72	1.43–1.64	1.53–1.83	1.42–1.57	1.45	1.28–1.72	1.32	1.40–1.70	1.33/1.45	1.13–1.26	1.04–1.15	1.13/1.19
	SPexp-1:MSexp-3	1.00–1.16	0.97	0.95–1.16	1.03–1.07	1.02–1.11	0.86–1.17	1.03–1.29	1.07	0.97–1.14	1.00	1.00–1.17	1.10/1.22			
	SPexp-1:ODSexp-3	0.90–1.13	0.88	0.92–1.10	0.88–0.95	0.90–1.18	0.88–1.15	0.89–1.15	0.91	0.82–1.17	0.85	0.85–0.94	0.97/1.03	0.94–1.13	0.93–1.00	0.89/0.96
L ratio spines on P3exp	SPexp-1:SPexp-2	1.05–1.38	1.14	1.10–1.22	1.16–1.30	1.14–1.35	1.00–1.37	1.03–1.43	1.03	1.06–1.21	1.10/1.13	1.07–1.26	1.42/1.44	1.14–1.26	1.15–1.30	1.24/1.25
	SPexp-1:PSexp-3	0.85–1.11	0.89	0.89–1.00	0.97–1.06	0.93–1.03	0.83–1.10	0.97–1.03	0.94	0.88–1.00	1.10	0.83–0.85	1.17	1.07–1.31	1.15–1.20	1.14/1.35
	SPexp-1:ODSexp-3	0.83–1.03	0.89	0.87–1.05	0.82–1.00	0.83–0.95	0.66–0.97	0.94–1.11	0.83	0.77–0.94	0.91/0.92	0.81–0.83	1.06/1.10	1.00–1.06	1.00–1.08	1.07/1.09
L ratio spines on P4exp	SPexp-1:SPexp-2	0.90–1.18	0.92/1.02	1.01–1.12	0.86–1.13	0.94–1.06	0.85–1.25	0.96–1.17	0.96	0.88–1.21	1.04	1.05–1.19	1.04	1.04–1.08	1.05–1.28	0.81/0.83
	SPexp-1:PSexp-3	0.82–1.07	0.73/0.90	0.87–1.06	0.80–0.93	0.84–0.97	0.86–0.93	0.87–1.08	0.86	0.75–0.97	0.92	0.82–1.02	1.10	1.00–1.27	1.00–1.17	0.94/1.14
	SPexp-1:ODSexp-3	0.72–0.92	0.69/0.79	0.83–0.97	0.70–0.93	0.78–0.94	0.72–1.04	0.79–1.00	0.78	0.65–0.88	0.76	0.74–0.78	0.91/0.93	0.81–0.93	0.78–1.00	0.81/0.83
L ratio spines on P2enp-3	ODS:DS	0.45–0.63	0.51/0.56	0.43–0.57	0.49–0.58	0.52–0.55	0.50–061	0.45–0.59	damaged	0.42–0.53	0.47/0.50	0.45–0.55	0.53/0.56	0.42–0.51	0.50–0.55	0.51
L ratio spines on P3enp-3	OSDS:DS	0.39–0.56	0.42	0.37–0.48	0.35–0.53	0.47–0.53	0.43–0.54	0.39–0.56	0.42	0.34–0.52	0.38/0.40	0.32–0.43	0.43	0.38–0.43	0.37–0.41	0.37/0.41
	ODS:DS	0.44–0.64	0.42/0.51	0.43–0.56	0.42–0.55	0.48–0.54	0.42–0.51	0.41–0.55	0.45	0.39–0.50	0.40/0.46	0.42–0.51	0.51/0.52	0.42–0.44	0.42–0.50	0.45/0.51
L ratio spines on P4enp-3	OSDS:DS	0.30–0.49	0.38	0.30–0.48	0.28–0.45	0.41–0.47	0.30–0.47	0.32–0.43	0.37	0.25–0.33	0.27/0.34	0.25–0.32	0.34	0.35–0.38	0.34–0.38	0.26/0.31
	ODS:DS	0.36–0.50	0.42	0.32–0.48	0.33–0.52	0.42–0.51	0.39–0.53	0.36–0.48	0.43	0.29–0.38	0.36/0.38	0.28–0.41	0.42	0.39–0.41	0.40–0.43	0.34/0.37

* = data from the left/right legs of one specimen; if there is only one datum, it means that the values are the same or only one side was measured.

**Male (Figs 5, 16E, Tables 3, 4).** Body length range 298–331 µm in Pacific specimens (Table 3). Sexual dimorphism in antennule, maxilliped, P6, and in genital segmentation, slight modification in setal length of P5.

P5-bearing somite with paired row of midventral spinous processes (Fig. 5D), variable in number, generally two or three processes.

Caudal rami (Fig. 5A, D, F) with length to width ratio 1.7–2.0 measured along inner margin and 2.3–2.7 measured along outer margin (Table 3). Caudal setae with variations in proportional lengths of caudal setae III:II and setae IV:III as given in Table 3, similar to female. CR seta II ornamented with single long spinule in some specimens (Fig. 16E), not noted for specimens from Red Sea.

Dorsal surface of genital somite ornamented with pattern of minute denticles or spinules (Fig. 5D), which are less distinct than in Red Sea specimens (Böttger-Schnack 2003: fig. 5D), ventral surface with spinule pattern on anterior part (Fig. 5F) not observed in Red Sea specimens (Böttger-Schnack 2003: fig. 5E). Surface of genital flaps covered with several rows of strong denticles or spinules (Fig. 5E, F), few denticles also observed on inner distal part (Fig. 5D) not observed in Red Sea specimens (Böttger-Schnack 2003: fig. 5D).

Antennule (Fig. 5G) 4-segmented, armature formula: 1-[3], 2-[8], 3-[4], 4-[9+2ae+(1+ae)], aesthetascs very small and slender, segment 4 with small middle aesthetasc close to seta present, which is not discernible in the female. Ornamentation as figured.

Antenna (not figured) with variation in length to width ratio of distal endopod segment similar to female (Table 3).

Maxilliped (Fig. 5B, C) 3-segmented, comprising syncoxa, basis and 1-segmented endopod, armature and ornamentation as figured. Basis with only one long seta within longitudinal cleft, corresponding to distal seta in female, proximal seta absent (Fig. 5C). Endopod represented by long curved claw, tip of claw without hyaline apex.

Swimming legs 1–4 with armature and ornamentation as in female. Variability in length ratios of outer spine on exp-1 relative to outer spines on exp-2 and exp-3 of P2–P4, and length ratios of outer subdistal spine and outer distal spine relative to distal spine on enp-3 of P2–P4 given in Table 4, not significantly different from female.

P5 (Fig. 5E, F) exopod with general shape and armature as in female; exopodal seta and outer basal seta somewhat shorter than in female.

P6 (Fig. 5F) represented by posterolateral flap closing off genital aperture on either side, ornamented as described above, posterolateral corners well discernible in dorsal aspect (Fig. 5A, D).

###### Remarks.

Böttger-Schnack (2003) provided a comprehensive redescription of *S. ivlevi* from the Red Sea and various other regions and included a detailed discussion of Shmeleva’s descriptions of the species in 1966 (original account) and in 1969 and of that record by Malt (1982). Therefore, these papers are not further discussed in the present paper and the data presented by Böttger-Schnack (2003) were mainly used as a reference for comparison with the Pacific specimens. However, one detail of Shmeleva’s original illustration is noteworthy, as the shape of the distal endopod segment on the antenna is much more slender in both sexes (Shmeleva 1966: fig. 1.4; 1969: figs 3d, 4c) than figured in Böttger-Schnack’s account for the robust form of the female (2003: fig. 3A). In specimens of both female form variants from the Pacific the distal endopod segment of the antenna appears to be relatively longer and more slender than figured for the Red Sea specimens, showing a range of variation in length to width ratio of 3.0–3.9 in Pacific (cf. Table 3), while this ratio is described as “about three times longer than wide” in the Red Sea (Böttger-Schnack 2003: 198). As the figure of the specimen from the Korea Strait (Fig. 6A) also shows a somewhat stronger reflexed orientation of the distal segment compared to the specimen from the equatorial Pacific (Fig. 3B), the length to width ratio may be underestimated. But the respective figure (Fig. 6A) does not give clear evidence about its actual length to width ratio, because the strongly reflexed orientation of the distal antennary segment makes it difficult to measure it from this figure.

Some other differences between our study and Böttger-Schnack’s redescription were detected in the presence of few long fine setules on the intercoxal sclerite of P1 in both sexes (Figs 4a, 16C), and the distinct ornamentation of the ventral anterior surface of the genital somite in the male (Fig. 5F). The first character mentioned has so far been found only in one other *Spinoncaea* species, *S. tenuis* (cf. Böttger-Schnack 2003: fig. 14A), and is recorded for *S. ivlevi* in the present account for the first time, but seemed to be variable, being present in most but not all specimens examined from the three locations in the Pacific (e.g., eight of eleven females and three of four males in the northwestern Pacific).

Additional or different ornamentation details found in the Pacific specimens of *S. ivlevi*, not mentioned and/or not figured by Böttger-Schnack (2003) included mainly details on the surface of elements such as on the maxilla (syncoxa with additional spinule pattern, Fig. 3E), or small details on setae, such as on the inner seta on the coxobasis of the antenna (Figs 3B, 6A) and on the middle element on the outer lobe of the maxillule (Figs 3D, 16B). These delicate ornamentation details can be discerned much better under a scanning electronic microscope as used in the present study than under a light microscope.

Despite the ornamentation differences between the redescription (Böttger-Schnack 2003) and the present account, specimens from the equatorial and temperate Pacific Ocean were regarded as conspecific with *S. ivlevi* because our specimens showed basic morphological characters of *S. ivlevi*, such as:

1. the mandible showing the full set of 5 elements,
2. the length to width ratio of the caudal ramus,
3. the proportional lengths of caudal setae,
4. the shape of caudal seta IV, which is setiform and not dilated as in S. tenuis,
5. the shape and ornamentation of the female genital double-somite, and
6. the paired row of long setules on the anterior surface of the labrum.

In addition, the results of the molecular genetic analysis, which are presented, also supports this opinion, and is briefly discussed below.

Similar to the report from the Red Sea (Böttger-Schnack 2003), females of *S. ivlevi* exhibited two form variants in the equatorial northeastern and northwestern Pacific. Taking into consideration the variability of morphological characters of the two variants as examined in the present account (Tables 3, 4), the following differences between the two female forms reported by Böttger-Schnack (2003: 204) could be confirmed for specimens from the Pacific: (1) the length to width ratio of the anal somite, which is larger in the elongate form (1.2–1.4) than in robust form (1.0–1.1), (2) the length ratio of prosome to urosome which is smaller in the elongate form (1.3–1.4) than in the robust form (1.5–1.7), and (3) the shape of the genital double-somite, which shows a stronger degree of tapering in the elongate form (Fig. 7A) as compared to the robust form (Fig. 2C). On the other hand, the difference between the two forms in the length to width ratio of the genital double-somite indicated in Böttger-Schnack´s study (2003: 204) was not confirmed, because the respective values in the Pacific specimens overlapped. (Table 3). Also, the variability of the length to width ratio of the caudal ramus is similar for both variants, and the range of values of proportional spine lengths of endopodal and exopodal spines on P2–P4 overlap between the two forms, including the values of these spines calculated from the robust form in Böttger-Schnack (2003: fig. 4B–D). The P5-bearing somite of the elongate form from the equatorial Pacific exhibits one pair of weakly developed ventrolateral lobes (Fig. 7B, C), which is not mentioned in the descriptive text of Böttger-Schnack (2003), but was shown in her fig. 6b. In the robust form, these lobes were not observed in specimens from the two locations in the equatorial Pacific areas (cf. Fig. 2E), but were weakly pronounced in specimens from the Korea Strait (Fig. 4D, arrowed).

In the Pacific, individual variation between specimens was found e.g., in the number of midventral spinous processes on the P5-bearing somite, either two or three in both sexes, and some individuals also had different numbers between left and right side (Fig. 2E). It is common that there is no fringe of long setules on outer margin of proximal endopod segment of P4 in *S. ivlevi*, but in some individuals this fringe was present (not figured). Furthermore, individual variation in ornamentation appeared (1) in the caudal seta II in some individuals, ornamented with a single long spinule in both sexes, (2) in the ornamentation on the dorsal anterior surface of the genital double-somite of females (cf. Fig. 2C), not observed in all specimens. One of the robust females from the Korea Strait (Fig. 6A–D) showed intraspecific variation in the outer distal part of the first endopod segment of the antenna with broad and more numerous spinules (arrowed in Fig. 6a), in additional setules on the anterior surface of the labrum (Fig. 6C), in one of the spinules on the inner margin of the syncoxa of the maxilla being relatively long (arrowed in Fig. 6B), and in the weak development of the ventrolateral lobes on the P5-bearing somite (Fig. 6D).

A number of morphological aberrations found in some specimens of *S. ivlevi* were summarized in Table 5. In the northwestern Pacific Ocean, three out of eleven robust female form variants and one out of four males showed abnormalities. Two aberrant specimens were ornamented with a patch of long setules on the anterior part of the cephalosome (Fig. 7D, E) and the other one robust female was ornamented with a very long setule on the dorsal anterior surface of the genital double-somite. In the northeastern Pacific Ocean, three out of six elongate females showed a pair of extremely long setules on both sides of the P4-bearing somite in ventral view (e.g., Fig. 7B), and one of them had also two extremely long setules on the ventral anterior surface of the genital double-somite (Fig. 7B). In the Korea strait, one robust female showed an atypical spine count on the right leg of P2, with only two outer spines on P2exp-3 [typical for the spine count on P2exp-3 of *S. humesi*] and with an inner setal count of four setae instead of five setae, while the armature on the right leg was normal. One male from the Korea strait showed imperfect and/or flawed segmentation of endopod segments on the antenna, and the distal part of abnormal distal segment has aberrant four setae.

**Table 5.** The morphological abnormalities of *S. ivlevi* from three locations in the Pacific Ocean Abbreviations: RF1, RF2 = female robust form; EF1, EF2, female elongate form; M = male; for abbreviation of locations see Tables 3, 4.

Specimens	Figure	Morphological abnormalities or variation
WP-RF1	Fig. 7D, E	- a patch of long setules on the anterior part of the cephalosome - the reduced length on both enp-3 of P1 with modified setae
WP-RF2	Fig. 7D, E	- a patch of long setules on the anterior part of the cephalosome
WP-RF3	not figured	- a long setule (or a seta) on the dorsal anterior surface of the genital double-somite
WP-M1	not figured	- reduced length of both enp-3 of P4 with modified spines and OSDS absent
EP-EF1	Fig. 7B, C	- two pairs of extremely long setules on both sides of the P4-bearing somite in ventral view
		- two extremely long setules on the ventral anterior surface of the genital double-somite
EP-EF2 and EP-EF3	Fig. 7B	- two pairs of extremely long setules on both sides of the P4-bearing somite in ventral view
KS-RF1	not figured	- one inner seta and one outer spine absent on the right exp-3 of P2
KS-M1	not figured	- abnormal shape of distal endopod segment on the antenna with aberrant setae

##### Spinoncaea tenuis

Taxon classificationAnimaliaCyclopoidaOncaeidae

Böttger-Schnack, 2003

C353F0A5-613C-56FD-8BC1-164C838FCF7C

**Figure 8.**
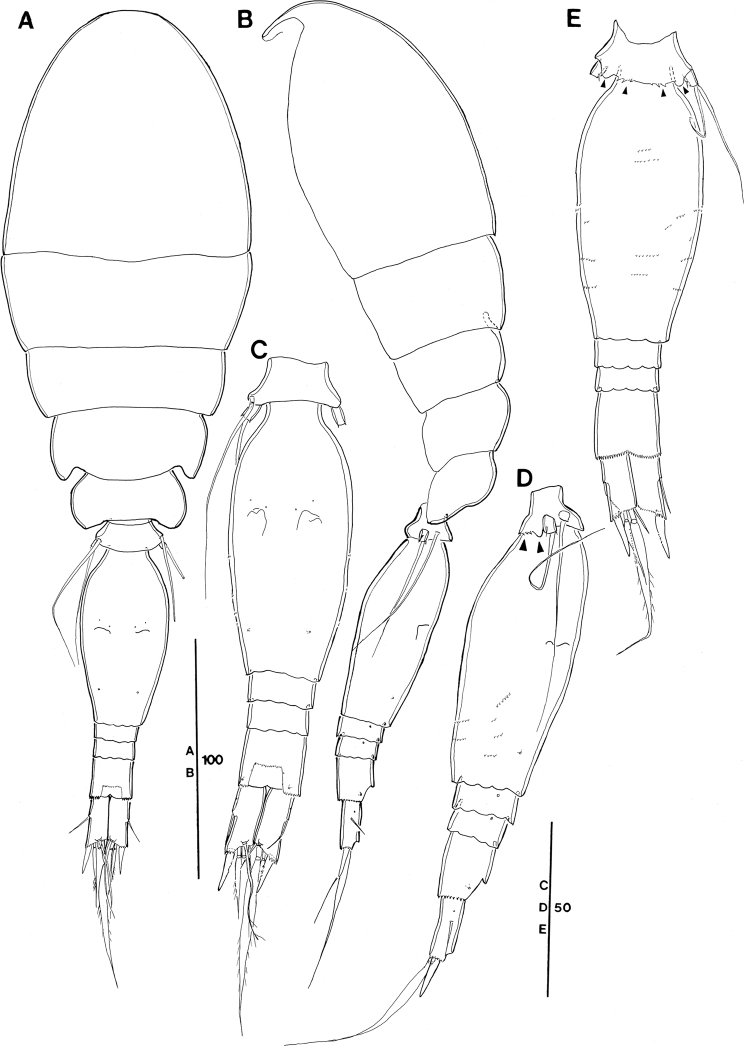
*Spinoncaea tenuis* Böttger-Schnack, 2003, female (northeastern equatorial Pacific) **A** habitus, dorsal (outer basal seta and exopodal seta of P5 on right side damaged, caudal seta V on left side missing) **B** habitus, lateral **C** urosome, dorsal (outer basal seta and exopodal seta of P5 on right side missing, caudal seta V on left side missing) **D** urosome, lateral, midventral spinous processes and ventrolateral lobe arrowed **E** urosome, ventral, midventral spinous processes and ventrolateral lobes arrowed (caudal setae IV and VI on left side omitted and seta V missing). Scale bars in μm.

**Figure 9.**
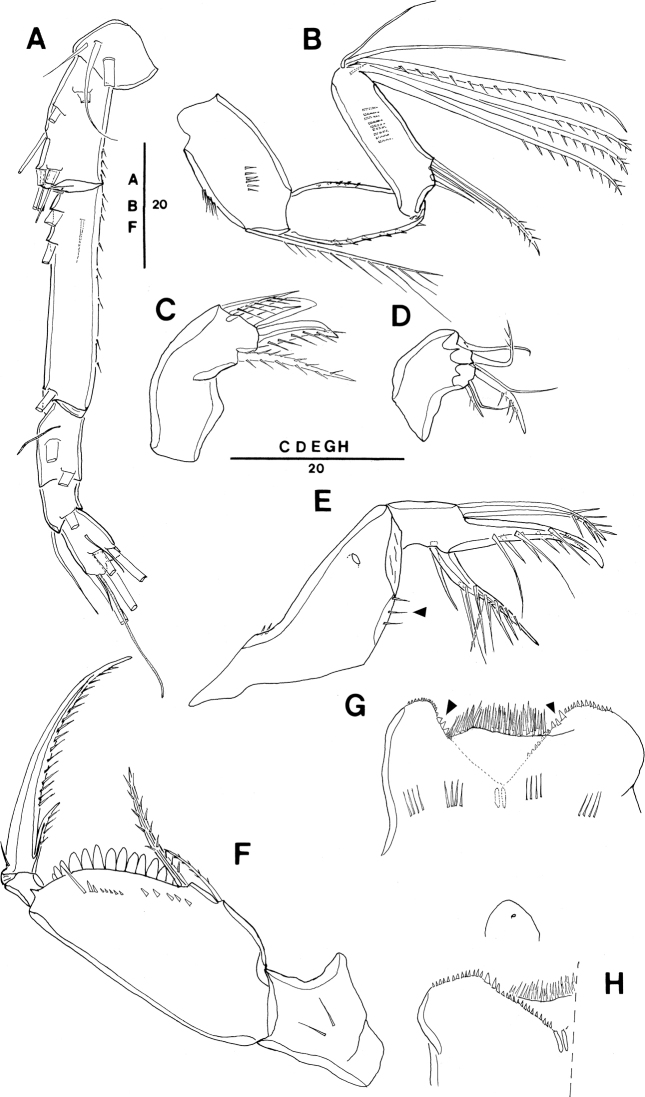
*Spinoncaea tenuis* Böttger-Schnack, 2003, female (northeastern equatorial Pacific) **A** antennule **B** antenna **C** mandible **D** maxillule **E** maxilla, arrows indicating spinules **F** maxilliped, anterior **G** labrum, anterior, arrows indicating three marginal teeth **H** labrum, posterior. Scale bars in μm.

**Figure 10.**
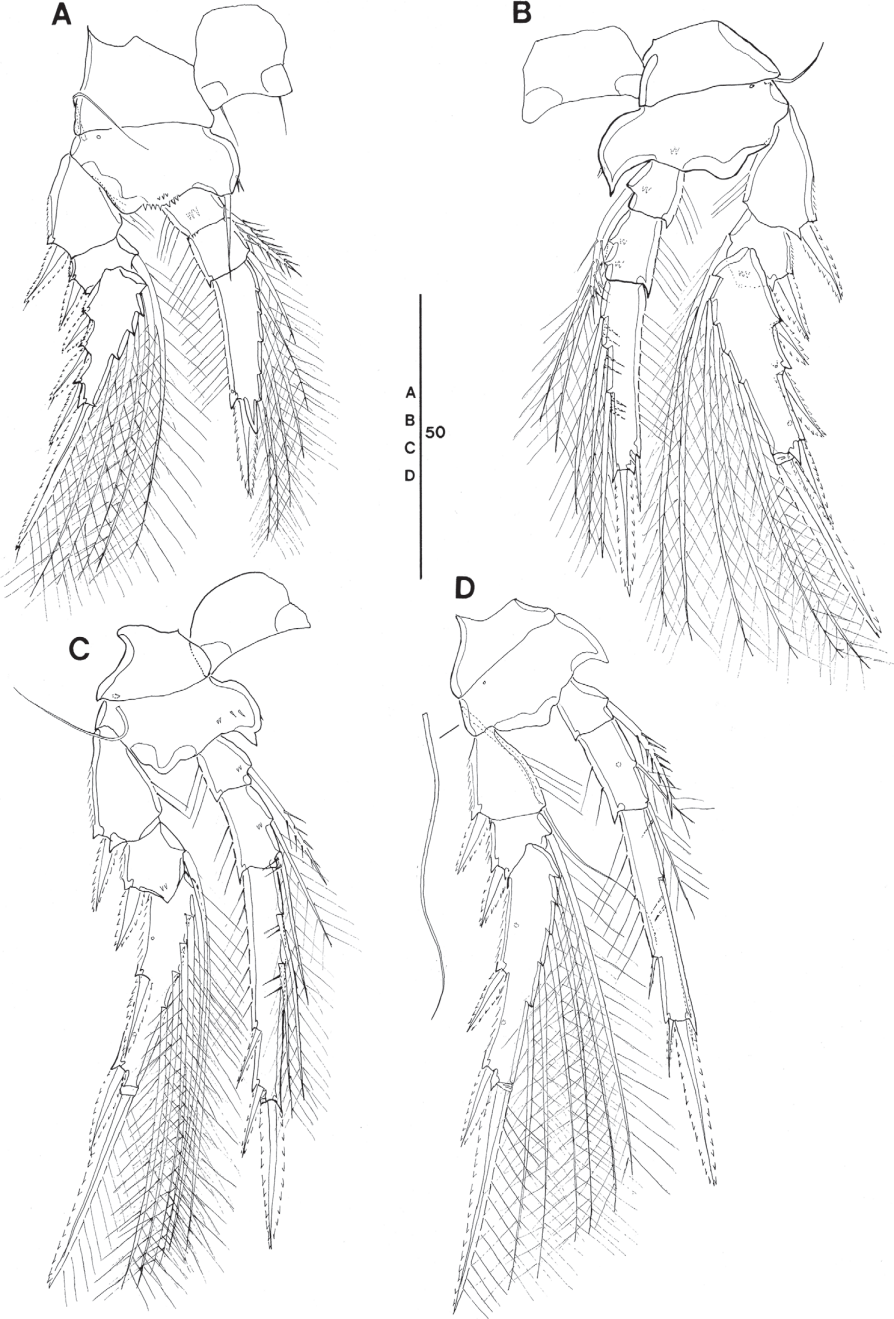
*Spinoncaea tenuis* Böttger-Schnack, 2003, female (northeastern equatorial Pacific) **A**P1, anterior **B**P2, anterior **C**P3, posterior **D**P4, anterior, intercoxal sclerite not figured, seta on basis figured separately. Scale bars in μm.

**Figure 11.**
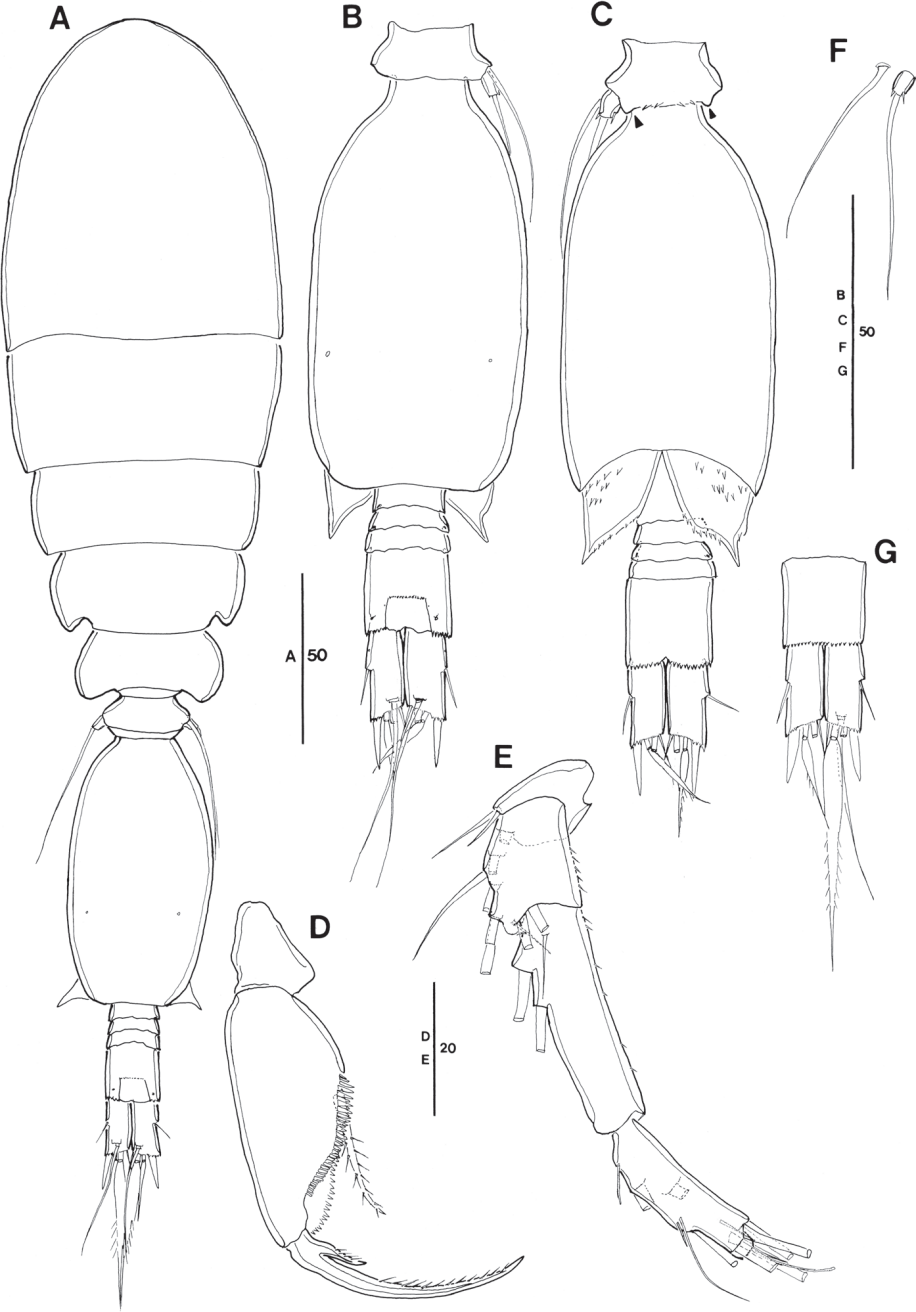
*Spinoncaea tenuis* Böttger-Schnack, 2003, male (northeastern equatorial Pacific) **A** habitus, dorsal (outer basal seta on left side of P5-bearing somite missing) **B** urosome, dorsal (P5 and the outer seta of P5-bearing somite on left side missing, caudal seta V on both sides missing) **C** urosome, ventral, ventrolateral lobes on P5-bearing somite arrowed (P5 and the outer seta of P5-bearing somite on left side missing, caudal seta V on both sides missing) **D** maxilliped, anterior **E** antennule **F**P5 exopod and outer basal seta, lateral **G** Anal somite and caudal ramus of another specimen, ventral. seta IV on left side and seta V on right side omitted. Scale bars in μm.

Figs 8 , 9 , 10 , 11

Spinoncaea tenuis Böttger-Schnack, 2003: 215–225, figs 12–16 (Red Sea, Mediterranean, Arabian Sea, Pacific Ocean).

###### Material examined.

(1) Northeastern Pacific (a) 10°30'N, 131°20'W (EP-1), 21 August 2009: One female (habitus of *S. tenuis* female in Fig. 8A, B) and one male (habitus of *S. tenuis* male in Fig. 11A) undissected on H-S slide, respectively. Five females and two males dissected on several slides, respectively. Three females dissected on H-S slide, respectively. Six dissected females (NIBRIV0000882784–882789) and one dissected male (NIBRIV0000882790) and one undissected female (NIBRIV0000882782) and one undissected male (NIBRIV0000882783) on respective H-S slide were deposited in the NIBR. (b) 9°52'1.38"N, 131°45'38.28"W (EP-2), 19 March 2019. Two undissected females and two undissected males in alcohol vial (NIBRIV0000882791) were deposited in the NIBR. (2) Northwestern Pacific, 13°20'3.42"N, 144°20'2.7"E (WP-2), 4 April 2016. One undissected male in alcohol vial (NIBRIV0000882792) was deposited in the NIBR. (3) Korea Strait, 33°44'50.50"N, 128°15'39.02"E (KS), 7 October 2008: One female (NIBRIV0000882793) and one male (NIBRIV0000882794) dissected on H-S slide, respectively. All dissected specimens and one undissected female (in alcohol, NIBRIV0000882795) were deposited in the NIBR.

###### Description.

**Female (Figs 8–10, Tables 3, 4).** Body length in lateral view (telescoping of somites not considered) (Fig. 8B) 320–355 µm in Pacific specimens (Table 3), somewhat larger than in the Red Sea (280–300 µm, Böttger-Schnack 2003: 215).

Prosome 1.7 × length of urosome, excluding caudal rami, 1.5 × urosome length including caudal rami in specimens figured (Fig. 8B), calculated by not correcting for the telescoping of somites. Variation of prosome to urosome length (including CR) 1.3–1.7 in Pacific specimens (Table 3), single value from Korea Strait smallest. The respective values provided for Red Sea specimens (1.5 incl. CR; Böttger-Schnack 2003: fig. 12A, calculated by not correcting for telescoping of somites) are within the range of values from the Pacific.

P5-bearing somite with paired midventral spinous processes variable in number (two or three processes) and one pair of ventrolateral lobate processes (arrowed in Fig. 8D, E). Variation in number of midventral spinous processes was not mentioned for Red Sea specimens and ventrolateral lobes were not described, but are vaguely discernible from Böttger-Schnack (2003: fig. 12I).

Genital double-somite (Fig. 8C, D, E) 2.1 × as long as maximum width in specimen figured (measured in dorsal aspect) and ~ 2.1 × as long as postgenital somites combined; variation in length to width ratio 1.8–2.3 in Pacific specimens (Table 3), respective values from Red Sea fall within this range. Largest width measured at 2/5 the distance between anterior and posterior margin, similar to Red Sea specimens, where it is “about halfway”. Ventral surface with few rows of minute spinules in some specimens (Fig. 8E), difficult to discern; this ornamentation was not mentioned for Red Sea specimens. Paired genital apertures located dorsally at about same position as in Red Sea specimens, armature difficult to discern. Weakly pronounced undulate hyaline frill on posterior margin of genital double-somite and postgenital somites and pore pattern as figured (Fig. 8D, E).

Anal somite (Fig. 8C) length to width ratio ranging between 1.1–1.3 (Table 3), ornamentation as figured (Fig. 8C, D, E).

Caudal ramus (Fig. 8A, C) length to width ratio 1.8–2.5 measured along inner margin and 2.3–3.0 measured along outer margin (Table 3). Caudal seta III ornamented with few minute spinules along medial margin (Fig. 8C), not observed in Red Sea specimens. Length ratio between seta IV and III 1.4–2.3 (Table 3), seta IV unipinnate, not bipinnate as in Red Sea specimens (Böttger-Schnack 2003: fig. 12C).

Antennule 6-segmented (Fig. 9A). Armature formula and ornamentation as for *S. ivlevi*.

Antenna 3-segmented, armature and ornamentation as figured (Fig. 9B). Distal endopod segment with length to width ratio 3.3–4.1 in Pacific specimens (Table 3), seta II longer than seta I (as illustrated for Red Sea specimens, Böttger-Schnack 2003: fig. 13A, but erroneously described as being “shorter than seta I” in text on p. 217).

Labrum with ornamentation as figured (Fig. 9G, H). including difference to *S. ivlevi* in (1) size of three marginal teeth along distal (ventral) margin on each lobe (arrowed in Fig. 9G) being somewhat smaller than in *S. ivlevi*, and (2) presence of two paired rows of long setules on anterior surface (Fig. 9G), not only a single paired row as in *S. ivlevi*.

Mandible with armature and ornamentation as figured. (Fig. 9C), small element D on gnathobase absent, as typical for *S. tenuis* (cf. Böttger-Schnack 2003: 218, fig. 13D).

Maxillule (Fig. 9D) similar to *S. ivlevi*, except for middle element on inner lobe naked.

Maxilla (Fig. 9E) with additional ornamentation on surface of syncoxa (arrowed in Fig. 9E), not reported earlier for Red Sea specimens.

Maxilliped as figured (Fig. 9F), surface of syncoxa ornamented with few spinules (arrowed in Fig. 9F), which was not recorded for Red Sea specimens.

Swimming legs 1–4 (Fig. 10A–D), with armature as in *S. ivlevi* (Table 2). Intercoxal sclerites of P1 ornamented with paired long, fine setules (but only one paired setule shown in Fig. 10A), which are difficult to discern. Ornamentation on inner portion of basis in P1–P3 as figured (Fig. 10A–C).

Exopods with variability of proportional spine lengths given in Table 4, respective values from the Red Sea fall within this range (Böttger-Schnack 2003: fig. 14A–D), except for the proportional lengths of outer spines on P3, which are larger in Pacific specimens than in the Red Sea specimens. Distal spine slightly longer than (P1) or almost equal in length (P2–P4) to distal exopod segment, similar to Red Sea specimens (Böttger-Schnack 2003: fig. 14A–D).

Endopods. Length ranges of outer subdistal spine and outer distal spine relative to distal spine on P2–P4enp-3 as given in Table 4 generally similar to Red Sea specimens (Böttger-Schnack 2003: fig. 14A–D).

P5 (Fig. 8C, D, F) exopod 1.7 × longer than wide, armature and ornamentation as figured.

P6 (Fig. 8C) as figured, possibly armed with a short spinule, which is difficult to discern.

**Male (Fig. 11, Tables 3, 4).** Body length 292–325 µm (Table 3). Sexual dimorphism in antennule, maxilliped, P6, and in genital segmentation, slight modification in setal length of P5. Pore pattern on prosome not discerned.

P5-bearing somite with paired row midventral spinous processes variable in number as in female and one pair of ventrolateral lobate processes (Fig. 11C).

Caudal rami (Fig. 11A, B, C, G) with length to width ratio 1.9–2.4 measured along inner margin and 2.2–2.8 measured along outer margin (Table 3), ornamentation as figured (Fig. 11D). Caudal setae with proportional lengths as in female, variation in length ratios as given in Table 3.

Antennule (Fig. 11E) 4-segmented, armature as for *S. ivlevi*.

Antenna (not figured) with variation in length to width ratio of distal endopod segment similar to female (Table 3).

Maxilliped (Fig. 11D) 3-segmented, armature and ornamentation as figured.

Swimming legs 1–4 with armature and ornamentation as in female. Variability in proportional spine lengths on rami given in Table 4, values of equatorial Pacific fall within the range of females, but proportional lengths of exopodal spines on P2 and P4 from Korea Strait larger than those of females.

P5 (Fig. 11F) with exopodal seta and outer basal seta somewhat shorter than in female.

P6 (Fig. 11C) with ornamentation pattern as figured.

###### Remarks.

Böttger-Schnack (2003) reported two variants of female *S. tenuis* which differed in geographical distribution. The typical form appeared in the entire Red Sea and in the northern Arabian Sea, while the elongate form was found in the Mediterranean Sea and in the NW Pacific (Kuroshio Extension); specimens from the NE Pacific (Monterey), on the other hand, showed intermediate values between both forms. In the present study, females from the NE equatorial Pacific also displayed intermediate values in morphological characters between the two forms of *S. tenuis*, which are as follows: (1) the length to width ratio of the genital double-somite has a wide range (1.8–2.3), including values of both form types; (2) the position of the genital apertures is at 2/5 of distance from the anterior margin as in the elongate form (from the Adriatic Sea); (3) the basal seta on P4 is more similar to the typical Red Sea form, reaching as far as the middle of the distal exopod segment, whereas this seta is much longer in the elongate form (from the Adriatic Sea), reaching beyond the tip of the distal spine on the exopod segment (cf. Böttger-Schnack 2003: fig. 16C); (4) the outer basal seta on P5 reaching as far as 4/5 the distance from the anterior margin of the genital double-somite in our Pacific specimens, but extending almost beyond the posterior margin of the genital double-somite in the elongate form (from the Adriatic Sea), (5) the length to width ratio of the caudal ramus measured along inner or outer margin in our specimens (1.8–2.5 or 2.3–2.9 ×, respectively) is larger than in the typical form from the Red Sea (1.9–2.0 or 2.1–2.3 ×) at least for ratio of the outer margin, and the range of values corresponds approximately to those of the elongate form from the Adriatic Sea (1.8–2.4 or 2.2–2.6 ×) and the NE Pacific (off Monterey, California) (2.1 or 2.4–2.7 ×) (Böttger-Schnack 2003: table 8).

In terms of ornamentation details, which are described for the typical form only, our Pacific specimens differed from the typical *S. tenuis* mainly by some details such as: (1) (1a) on the syncoxa of the maxilla and (1b) on the proximal element of the maxilliped; (2) short spinule(s) on the inner margin of bases on P2 and P3; (3) ornamentation with few minute spinules along the medial margin of CR seta III; and (4) variable number of midventral spinous processes on the P5-bearing somite.

Unlike the females, males of *S. tenuis* could not clearly be classified into form types in Böttger-Schnack’s account. When compared to the typical form from the Red Sea, specimens from the equatorial Pacific are similar in morphology, except for some minor differences including (1) the length to width ratio of the genital somite, which is longer than in our specimens (1.8–2.0 ×) than in the Red Sea specimens (1.7 ×), (2) the caudal rami (inner 1.9–2.2 ×, outer 2.2–2.6 ×) were slightly longer than in the Red Sea specimens (inner 1.9 ×, outer 2.3 ×), and (3) the length ratio of caudal setae VII and IV, respectively, with seta VII being 1.6–1.9 × longer than seta IV in the Pacific specimens, whereas seta VII is only 1.4 × the length of seta IV in the Red Sea specimens. Also, the number of paired midventral spinous processes on the P5-bearing somite differs, showing only two processes in the Pacific, as compared to three processes in the Red Sea specimens. However, as the male specimen from the Korea Strait also showed three paired processes (not figured), and differences among individuals of *S. tenuis* females (two or three processes) were apparent, this ornamentation seems to be due to individual variation, and cannot be regarded as a regional difference.

According to Böttger-Schnack (2003), some slight morphological differences occurred between males of *S. tenuis* from the Red Sea and the Adriatic Sea (e.g., the proportional lengths of the genital somite and the caudal rami), but the determination of an elongate male appeared to be ambiguous. In our case, the above mentioned two characters are intermediate between the typical form (from the Red Sea) and the elongate form (from the Adriatic Sea) and the range of these values could be perceived as a variation among individuals (cf. Table 3). However, the single male of *S. tenuis* from the Korea Strait (not figured) seemed to be similar to the elongate form from the Adriatic Sea, as it differed from specimens from the equatorial Pacific specimens in the following characters (Table 3): (1) smaller body length: 292 μm; (2) the genital somite being slightly longer than in the equatorial Pacific, with a length to width ratio of 2.0:1; (3) the length to width ratio of the caudal rami being greater/higher (inner 2.4 ×, outer 2.8 ×) than in the equatorial Pacific (Table 3); (4) the anal somite slightly longer than in the equatorial Pacific, 1.2 × longer than wide; and (5) the outer basal seta on P5 reaching the posterior margin of the genital somite. In summary, the observed variation of features for *S. tenuis* in the Pacific indicates that the previously described form types of this species are not clearly separated; however, distinct form types may occur due to regionally reduced ranges of variation in the morphological details described here.

The female of *S. tenuis* can easily be confused with the elongate form of female *S. ivlevi* from the Pacific Ocean, due to the shape of the genital double-somite. However, as Böttger-Schnack (2003) mentioned the distinction between *S. tenuis* and *S. ivlevi* elongate form from the equatorial Pacific are: (1) the number of elements on the mandible (four in *S. tenuis*, but five in *S. ivlevi* elongate form) and (2) the number of spinules patches on the anterior surface of the labrum (two pairs in *S. tenuis*, but one pair in *S. ivlevi* elongate form, generally). Further morphometric differences between females of the two species may be found in (3) the proportional lengths of caudal setae III: II, which is smaller in *S. tenuis* (1.0–1.5 ×) as compared to *S. ivlevi* elongate form (1.6–2.0 ×) and (4) the length ratio between the distal spine and distal exopod segment on P2–P4, with the distal spine being almost equal in length to the distal segment in *S. tenuis*, whereas the spine is shorter than the segment in *S. ivlevi* elongate form (esp. on P4) (Table 4). Further minor differences between the two species are found in the patterns of the ornamentation on the ventral surface of the genital double-somite, as the elongate form of *S. ivlevi* (Fig. 7B) has a larger number of spinular rows than *S. tenuis* (Fig. 8E) and the ornamentation on the inner margin of caudal ramus, which is absent in *S. tenuis*, but is present in *S. ivlevi* elongate form.

##### Spinoncaea humesi

Taxon classificationAnimaliaCyclopoidaOncaeidae

Böttger-Schnack, 2003

FDA6A7FE-59DA-5F95-8FAC-11E5C585494E

**Figure 12.**
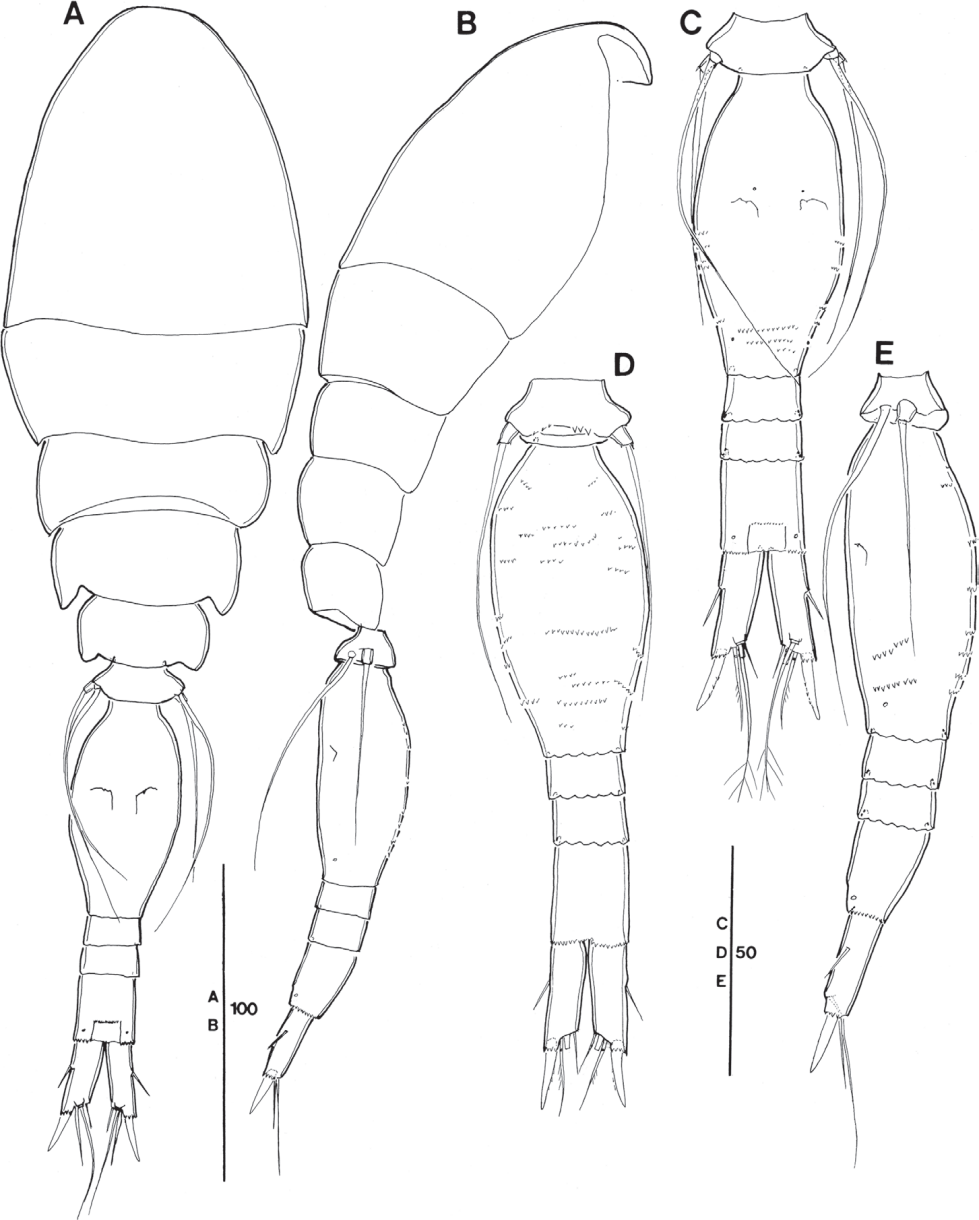
*Spinoncaea humesi* Böttger-Schnack, 2003, female (northeastern equatorial Pacific) **A** habitus, dorsal (caudal seta V missing on both sides) **B** habitus, lateral **C** urosome, dorsal, (caudal seta V missing on both sides) **D** urosome, ventral, (caudal seta V missing on both sides) **E** urosome, lateral. Scale bars in μm.

**Figure 13.**
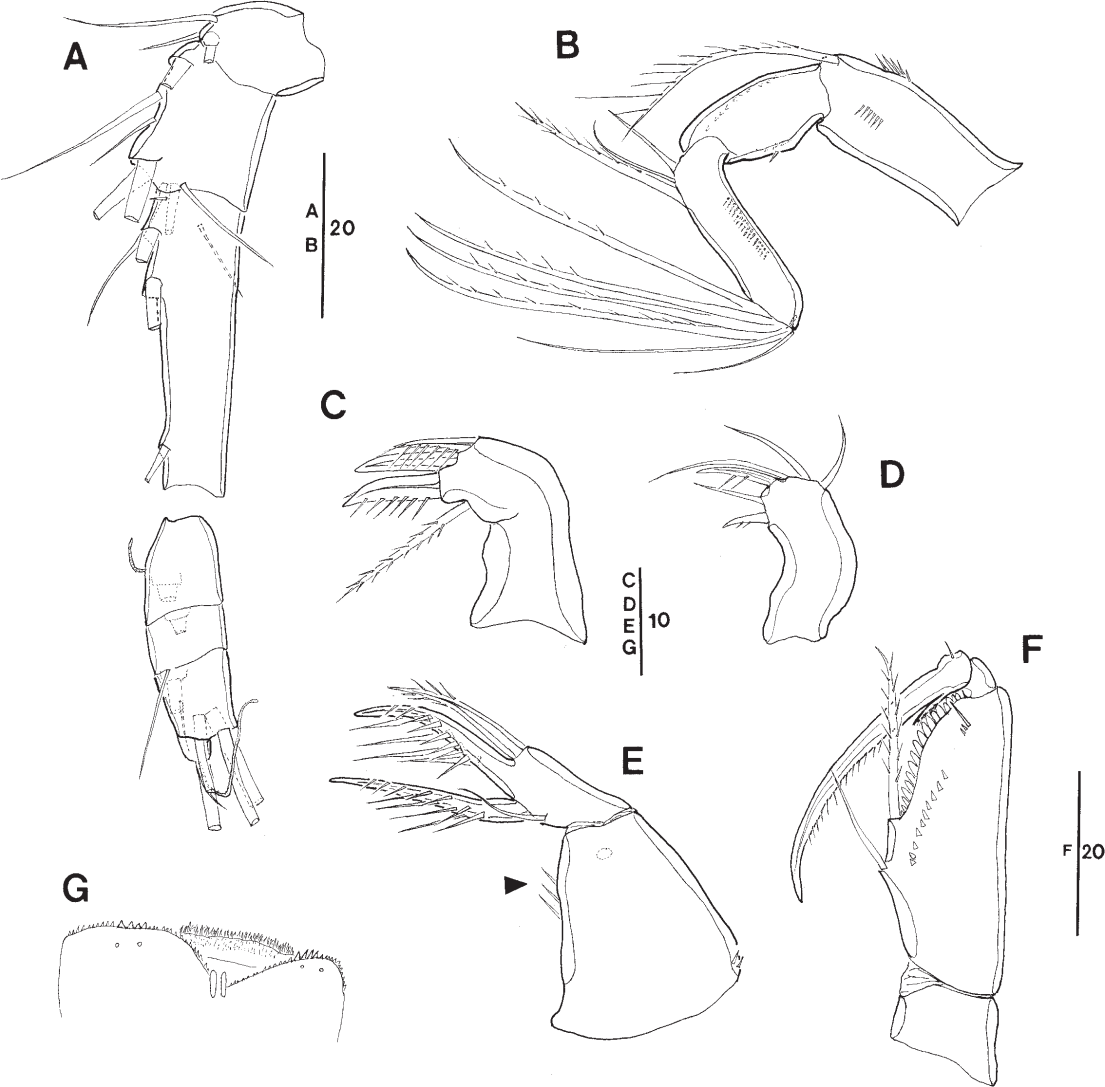
*Spinoncaea humesi* Böttger-Schnack, 2003, female (northeastern equatorial Pacific) **A** antennule (segments 4–6 drawn from another specimen) **B** antenna **C** mandible **D** maxillule **E** maxilla, arrows indicating spinules **F** maxilliped, anterior **G** labrum, posterior, showing some ornamentation on anterior side. Scale bars in μm.

**Figure 14.**
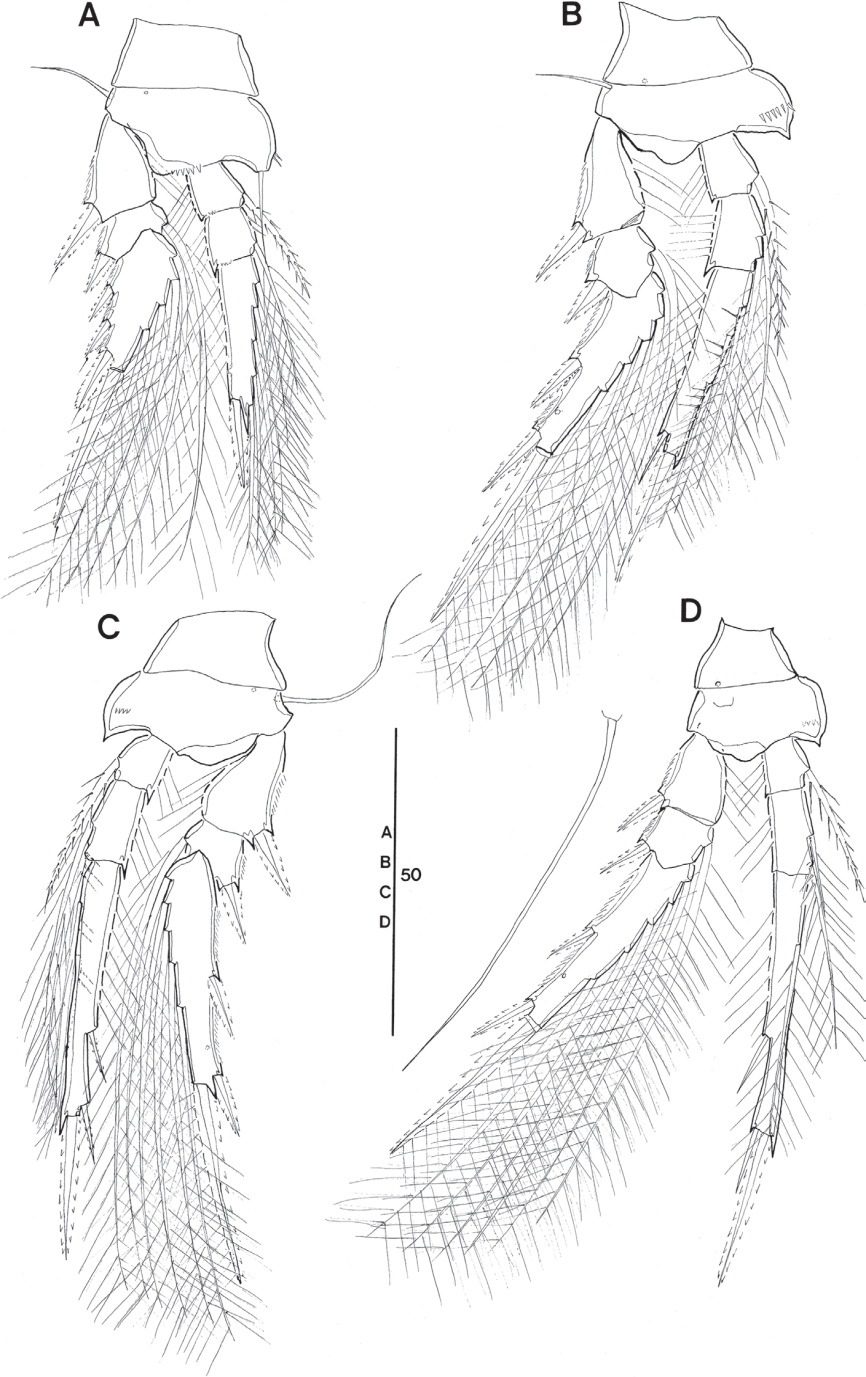
*Spinoncaea humesi* Böttger-Schnack, 2003, female (northeastern equatorial Pacific) **A**P1, anterior **B**P2, posterior **C**P3, anterior **D**P4, posterior, basal seta of another specimen figured separately. Scale bars in μm.

**Figure 15.**
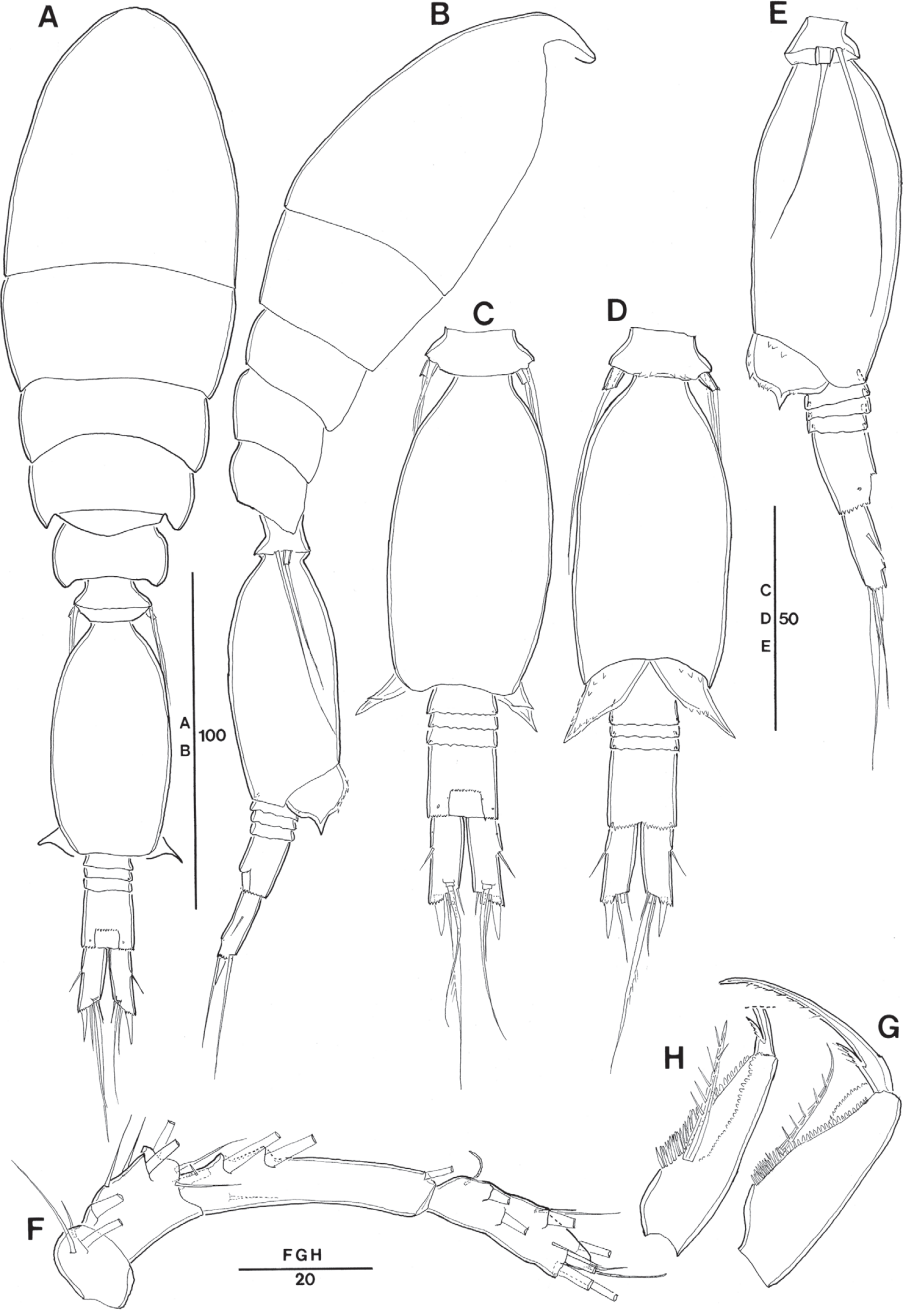
*Spinoncaea humesi* Böttger-Schnack, 2003, male (northwestern equatorial Pacific) **A** habitus, dorsal (caudal seta V on right side missing) **B** habitus, lateral **C** urosome, dorsal (caudal seta V on right side missing) **D** urosome, ventral (caudal seta V on right side missing) **E** urosome, lateral **F** antennule **G** maxilliped, anterior **H** maxilliped, middle. Scale bars in μm.

Figs 12 , 13 , 14 , 15

Spinoncaea humesi Böttger-Schnack, 2003: 208–215, figs 8–11 (Red Sea, Mediterranean, Indian and Pacific oceans).

###### Material examined.

(1) Northeastern Pacific, 9°52'1.38"N, 131°45'38.28"W (EP-2), 19 March 2019. Three females dissected on three or seven slides, respectively. Two dissected females (NIBRIV0000882796–882797) and two undissected females (in alcohol, NIBRIV0000882798) were deposited in the NIBR. (2) Northwestern Pacific (a) 13°23'46.44"N, 143°55'0.60"E (WP-1), 27 March 2016: Two males dissected on one or three slides, respectively. All dissected specimens (NIBRIV0000882799–882800) were deposited in the NIBR. (b) 13°20'3.42"N, 144°20'2.7"E (WP-2), 4 April 2016. One undissected male in alcohol (NIBRIV0000882801) was deposited in the NIBR. (3) Korea Strait, 33°44'50.50"N, 128°15'39.02"E (KS), 7 October 2008: One dissected male (NIBRIV0000882802) on H-S slide, and one undissected female and one undissected male in alcohol vial (NIBRIV0000882803) were deposited in the NIBR.

###### Description.

**Female (Figs 12–14, Tables 3, 4).** Body length in lateral view (telescoping of somites not considered) (Fig. 12B) 344–348 µm in northeastern Pacific (Table 3), somewhat larger than in the Red Sea (310–320 µm, Böttger-Schnack 2003: 208).

Prosome 1.7 × length of urosome, excluding caudal rami, 1.3–1.4 × urosome length including caudal rami (Fig. 12B, Table 3), for comparison with Red Sea see under “Remarks”. Integumental pores on prosome difficult to discern, not figured.

P5-bearing somite with three paired midventral spinous processes (Fig. 12D), no variation in number found (but see under “Male”).

Genital double-somite (Fig. 12C, D, E) 2 × as long as maximum width in specimen figured (measured in dorsal aspect) and ~ 1.5 × as long as postgenital somites combined; variation in length to width ratio given in Table 3, respective ratios from Red Sea specimens fit into this range; ornamentation of dorsal and ventral surfaces (Fig. 12D, E) as for Red Sea specimens, including weakly developed undulate hyaline frill on posterior margin of genital double-somite and postgenital somites, as well as absence of pores on lateral surface of postgenital somites (Fig. 12E).

Anal somite (Fig. 12C) with length to width ratio 1.2–1.3 (Table 3), similar to Red Sea, but slightly different from other areas reported in Böttger-Schnack's account (1.0–1.2:1, Böttger-Schnack 2003: table 7). One pair of secretory pores present dorsally near posterior margin (Fig. 12C), second pair reported for Red Sea specimens not discerned. Other ornamentation as figured (Fig. 12C–E).

Caudal ramus (Fig. 12A, C) 2.3–2.5 × longer than wide measured along inner margin and ~ 2.8–3.1 × longer than wide measured along outer margin (Table 3), range of variation similar to ratios reported for Red Sea and other regions (Böttger-Schnack 2003: table 7). Length ratios among setae II, III, and IV with ranges in Pacific specimens given in Table 3, Red Sea data fit into these ranges; seta V missing on both sides of specimen figured (measurements taken from undissected specimen as follows: seta V ~ 2.7 × longer than seta IV, 1.5 × longer than seta VII).

Antennule (Fig. 13A) with armature formula as for *S. ivlevi*. Ornamentation along inner non-setiferous margin of segments 2 and 3 absent, as specified for Red Sea specimens.

Antenna 3-segmented, armature and ornamentation as figured (Fig. 13B). Endopod segments ~ equal in length (but in Fig. 13B, the proximal endopod segment looks shorter than the distal one, due to its orientation on the slide); distal endopod segment ~ 4 × longer than wide, variation given in Table 3, Red Sea data fit into these ranges; armature and ornamentation as in *S. ivlevi*, except for seta II slightly longer than seta I (for numbering of elements see Fig. 3B).

Labrum with ornamentation as figured (Fig. 13G) including difference to *S. ivlevi* in size of four marginal teeth along distal (ventral) margin on each lobe being smaller than in *S. ivlevi*. Posterior face with two secretory pores on each lobe, which are difficult to discern. Anterior surface of labrum not observed in detail, but overlapping rows of fine spinules covering median concavity on anterior side visible from Fig. 13G.

Mandible with armature and ornamentation as figured (Fig. 13C), small element D on gnathobase absent, as typical for the species.

Maxillule (Fig. 13D) similar to *S. ivlevi*, except for middle element on outer lobe naked.

Maxilla with armature and ornamentation as figured (Fig. 13E), additional ornamentation on syncoxa in Pacific specimens arrowed in Fig. 13E.

Maxilliped with armature and ornamentation as figured (Fig. 13F), similar to Red Sea specimens, including small ornamentation details, such as proximal element on basis unornamented.

Swimming legs (Fig. 14A–D), with armature as in *S. ivlevi* except for spine count on distal exopod segment of P2, showing only two outer spines (Table 2). Intercoxal sclerites unornamented (missing in specimen figured). Surface of coxae and bases with sparse surface ornamentation as figured, outer basal seta on P4 very long, reaching as far as tip of distal exopod segment (Fig. 14D), as typical for the species.

Exopods with variability of proportional spine lengths in Pacific specimens given in Table 4, respectively values from Red Sea generally fit into these ranges, except proportional spine lengths on P2 larger than in the Red Sea specimens.

Endopods with length ranges of outer subdistal spine and outer distal spine relative to distal spine on P2 and P4 given in Table 4 generally similar to Red Sea specimens, except for outer distal spine relative to distal spine on P2 (0.45–0.51) and P3 (0.42–0.44) smaller than Red Sea (0.56 on P2 and 0.48 on P3, calculated from Böttger-Schnack 2003: fig. 10B, C).

P5 (Fig. 12C, E) with exopod 1.4 × longer than wide, shorter than in Red Sea (1.7:1; cf. Böttger-Schnack 2003: 208, fig. 8H, I), armature and ornamentation as figured.

P6 (Fig. 12C) as figured, armature (short spinule) difficult to discern.

**Male (Fig. 15, Tables 3, 4).** Body length 285–295 µm (Table 3). Sexual dimorphism in antennule, maxilliped, P6, and in genital segmentation, slight modification in setal length of P5. Pore pattern on prosome not discerned.

P5-bearing somite with paired midventral spinous processes variable in number (two or three processes) (Fig. 15D).

Caudal rami (Fig. 15A, C, D) with length to width ratio 2.1–2.5 measured along inner margin and 2.6–3.2 measured along outer margin (Table 3), [single value from Korea Strait larger than those from western equatorial Pacific,] respective values from Red Sea and other areas (Böttger-Schnack 2003: table 7) fit into this range. Ornamentation details as figured, similar to Red Sea specimens, including absence of surface ornamentation on genital somite (Fig. 15C, D).

Antennule (Fig. 15F) with armature as for *S. ivlevi*. Segments 2 and 3 without ornamentation.

Maxilliped (Fig. 15G, H) 3-segmented, syncoxa missing in specimen figured. Basis and endopod (claw) with armature and ornamentation similar to Red Sea specimen, including ornamentation detail on claw, with pinnules only along distal half of concave margin.

Swimming legs 1–4 with the value ranges in spine lengths on rami given in Table 4 not significantly different from female, except for the values of the endopodal spines on P4 from Korea Strait smaller than those of females.

P5 (Fig. 15B, E) with exopodal seta and outer basal seta shorter than in female, outer basal seta also much shorter than in Red Sea specimens (Böttger-Schnack 2003: fig. 11D–F).

P6 (Fig. 15D) with ornamentation as figured.

###### Remarks.

The morphology of both sexes of *S. humesi* from the Pacific agrees in most parts with the original description of the species by Böttger-Schnack (2003). As stated above, the Pacific specimens differ only in a few characters, such as in (1) a somewhat larger body size in the female and (2) the length ratio of the prosome to the urosome in the female, which appears to be slightly larger in the Pacific specimens (1.7:1 and 1.3–1.4:1, excluding and including caudal rami, respectively) as compared to the Red Sea specimens (1.5:1 excluding caudal rami and 1.2:1 including caudal rami, calculated from Böttger-Schnack (2003: fig. 8A). Note, that in the text of Böttger-Schnack (2003: 208) the proportions of the prosome to the urosome are given as 2.0:1 and 1.7:1, respectively, but these were calculated by a different method taking into account the telescoping of somites, while the telescoping of somites was not considered in the present study. Also, some additional ornamentations were found in the Pacific specimens, such as on the syncoxa of the maxilla of both sexes, the additional ornamentation on the inner portion of the basis of P2–P4 in our Pacific specimens or the number and size of spatulated spinules between proximal seta and articulation with endopod on the maxilliped in female, which are smaller and more numerous than in the specimen from the Red Sea.

The male of *S. humesi* from the Korea Strait agreed in almost all morphological characters with the specimens from the northwestern equatorial Pacific. But it exhibited individual variabilities in the length to width ratio of caudal ramus, the relative length ratio of caudal setae, and the length to width ratio of the genital somite (cf. Tables 3, 4). An additional variation in the male from the Korea Strait was found in the number of midventral spinous processes on the P5-bearing somite, with three paired processes (not figured), as in female, while in the male of the northwestern Pacific only two paired processes were found, as in the male from the Red Sea (Böttger-Schnack 2003: fig. 11E). The number of midventral spinous processes on the P5-bearing somite seems to be a common individual variation seen within both sexes among *Spinoncaea* species.

*Spinoncaea humesi* can easily be distinguished from the other two species of *Spinoncaea* by the number of spines on P2exp-3, showing two outer spines in *S. humesi*, but three spines in *S. ivlevi* and *S. tenuis*. Also, the outer basal seta of P5 is extremely long, extending beyond the posterior margin of the genital double-somite in the female, and the shape of genital double-somite is different, being barrel-shaped in *S. humesi*. Other additional characters for species segregation are not further mentioned in the present study because they are described in detail in the remarks section of *S. humesi* by Böttger-Schnack (2003: 214–215).

### Key to species of the genus *Spinoncaea*

**Table:** 

1	P2exp-3 with 2 outer spines; genital double-somite barrel-shaped in female	***S. humesi***
–	P2exp-3 with 3 outer spines; genital double-somite oval-shaped or elongate oval-shaped in female	**2**
2	Md with 5 elements; undulate or lobate hyaline frill at posterior margin of urosomites strongly pronounced; inner margin of caudal ramus with row of setules; modified seta III (spine) on caudal ramus very strong	***S. ivlevi***
–	Md with 4 elements; undulate hyaline frill at posterior margin of urosomites weakly pronounced; inner margin of caudal ramus naked; modified seta III (spine) on caudal ramus less strong	***S. tenuis***

The difference described for the mandible is not noticeable without difficult preparation of the mouthparts. Thus, this character is not included in the general identification key for Oncaeidae “OncIdent” of Böttger-Schnack and Schnack (2016–2021).

**Figure 16.**
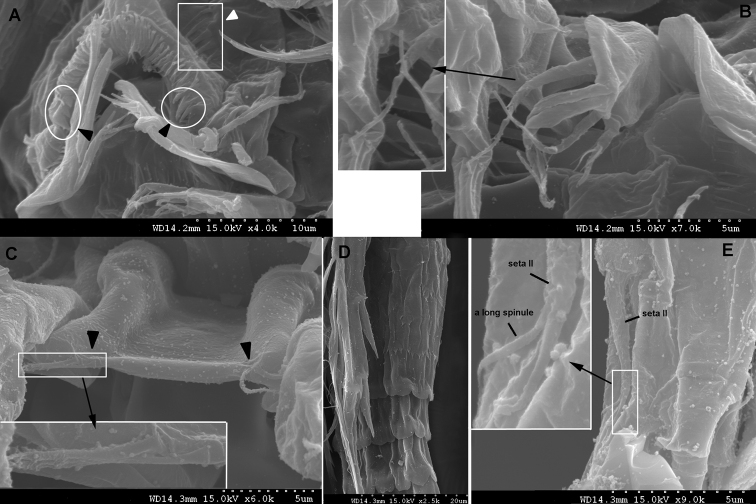
*Spinoncaea ivlevi* (Shmeleva, 1966), female, robust form (northwestern equatorial Pacific) **A** labrum, anterior, white arrow indicating setules (in square), black arrows indicating three marginal teeth (in circle) **B** maxillule, inset showing enlarged second element on outer lobe **C** intercoxal sclerite on P1, black arrows indicating ornamentation with long, fine setule, inset showing enlarged setules **D** posterior part of genital double-somite and postgenital somites showing undulate hyaline frill. *Spinoncaea ivlevi* (Shmeleva, 1966), male (northwestern equatorial Pacific) **E** caudal ramus seta II, inset showing enlarged seta II ornamented with a single long spinule.

### Molecular analysis

**Figure 17.**
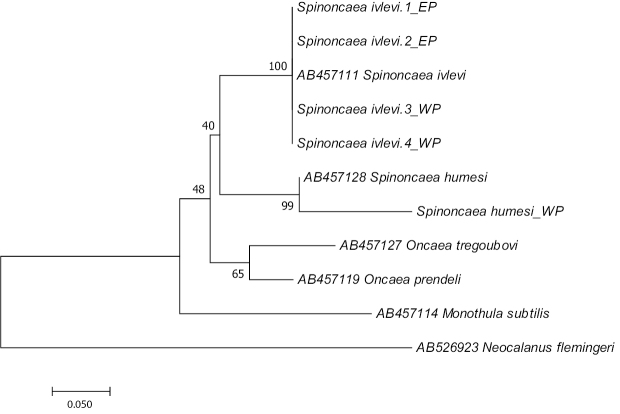
Maximum-likelihood tree from 12S sequences of two *Spinoncaea* species from the Pacific (WP, EP) and species of a clade including *Monothula* and the *ivlevi-tregoubovi* lineage as defined by Böttger-Schnack and Huys (2001). Sequences of compared species were obtained from GenBank as analysed by Böttger-Schnack and Machida (2011) and for the outgroup *Neocalanus flemingeri* by Machida and Tsuda (2010). Bootstrap values from 1000 replications.

All three species of *Spinoncaea*, including also the two forms of female *S. ivlevi*, were analyzed for mtCOI and 12S srRNA sequences during the present study, but only the 12S srRNA sequences of *S. ivlevi* (robust form) and of *S. humesi* were successfully obtained (Table 6). The mtCOI sequences, which allowed clear discrimination of even the most closely related species (Hill et al. 2001), were not successfully sequenced for the *Spinoncaea* species. The 12S srRNA sequences of *Spinoncaea* species obtained in this study can be found under the GenBank accession numbers MN714702–MN714706. The 12S srRNA sequences for individuals of *S. ivlevi* in the western and the northeastern Pacific Ocean were 100% identical to each other and were in concordance with *S. ivlevi* type sequence collected in the Mediterranean Sea (GenBank accession number AB457111; Böttger-Schnack and Machida 2011). The sequencing result of *S. humesi* from the Pacific was also 99% identical to the type sequence collected in the Mediterranean Sea (GenBank accession number AB457128; Böttger-Schnack and Machida 2011). Each node is strongly supported with high bootstrap values in 12S phylogenetic tree (Fig. 17). Within *S. ivlevi*, the *p*-distances were zero and the *p*-distances of between *S. ivlevi* and *S. humesi* were 0.16.

**Table 6.** Molecular analysis of three *Spinoncaea* species from the northeastern (NE) and northwestern (NW) equatorial Pacific: Collection region, number of individuals analyzed (N), number of DNA successfully isolated (n), and GenBank accession numbers of specimens successfully used for molecular analysis.

Species	N	n	Marker	GenBank accession no.	Collection region
*Spinoncaea ivlevi* (robust form)	14	2	12S	MN714703, MN714705	NE Pacific
	12	0	COI	–	
	5	2	12S	MN714704, MN714706	NW Pacific
*Spinoncaea ivlevi* (elongate form)	3	0	COI	–	NE Pacific
	6	0	12S	–	
	4	0	12S	–	NW Pacific
*Spinoncaea humesi*	3	1	12S	MN714702	NE Pacific
	3	0	12S	–	NW Pacific
*Spinoncaea tenuis*	10	0	12S	–	NE Pacific
	9	0	COI	–	
	10	0	12S	–	NW Pacific

## Discussion

*Spinoncaea* species are supposed to have a wide geographical distribution in warm-temperate and tropical areas, as they were described from various regions, such as the Mediterranean Sea (including the Adriatic Sea), the Red Sea, the Indian and the Pacific Oceans (Böttger-Schnack 2003). In the Pacific Ocean, however, earlier records mainly refer to higher latitudes (north of ~ 36°N), such as near Japan (Böttger-Schnack 2003; Nishibe and Ikeda 2004; Nishibe et al. 2009) or near California (Böttger-Schnack 2003). In the present study we add the open equatorial Pacific to their distribution, and they are first recorded in Korea’s surrounding waters.

Apart from the detailed morphological/taxonomical analysis and documentation (figures) of *Spinoncaea* species from the open equatorial Pacific, for the first time individual variation of numerous morphometric characters was analyzed for all three species, including proportions of body somites (e.g., anal somite, genital (double-)somite) and armature elements, such as the proportional lengths of endopodal and exopodal spines on the swimming legs, which have been found as limited but useful characters for differentiation between species of other oncaeid genera (e.g., *Triconia* Böttger-Schnack, 1999) (Heron et al. 1984; Heron and Bradford-Grieve 1995; Cho et al. 2013, 2017, 2019). The respective data obtained for *Spinoncaea* did in most cases not turn out to be useful for discrimination of the three species in this genus (Tables 3, 4). In some cases, however, the range of variation did not overlap among the species (e.g., the length ratio of distal exopod segment to distal spine on P4; cf. Table 4). Here a larger data set is required to clarify if these measures can be used for the differentiation of the species. Also, the data set will serve as a basis for comparative data with other oncaeid genera to understand the range of intraspecific variation of species in different parts of the world’s oceans.

Intraspecific variation among the three species was also found for ornamentation details, such as the number of midventral spinous processes on the P5-bearing somite in both sexes, which, however, is considered a commonly occurring variation in nature.

The morphological descriptions of *Spinoncaea* species by Böttger-Schnack (2003) included also details of ornamentation, but in Pacific specimens we found additional new ornamentation items which have not been reported in previous studies. For all three *Spinoncaea* species some additional ornamentation was found on the inner margin of the maxillary syncoxa, showing 3–5 long spinules. It is uncertain whether this has been overlooked in previous studies, as specimens from this area had not been described in detail in Böttger-Schnack’s study, or whether it newly emerged in specimens from the Pacific. Remarkably, a similar ornamentation on the syncoxa of the maxilla was also found in Pacific specimens of *Oncaea tregoubovi* Shmeleva, 1968 (unpublished thesis of Cho 2011: fig. 38F) and *tregoubovi*-group species (as *Oncaea* sp. 3 in Cho 2011: fig. 42E), based on copepod material collected from the same location in the northeastern equatorial Pacific (EP-1) as in the present study, and it was also found recently in *Oncaea prendeli* Shmeleva, 1966 from the southern Sea off Jeju Island (the East China Sea) (Cho et al. 2020). In earlier (re)descriptions of *O. tregoubovi* and *O. prendeli* from their type locality in the Adriatic Sea (Huys and Böttger-Schnack 2007), a corresponding ornamentation was not described (their figs 3F, 8F, respectively). Another ornamentation detail newly found in *S. ivlevi* from the Pacific was the ornamentation on the intercoxal sclerite on P1 in both sexes as well as the distinct ornamentation on the ventral surface of the genital somite in the male. In particular, the unique pattern in the male may be useful for distinguishing the males of the three *Spinoncaea* species. However, it is not easy to observe the ornamentation of these small sized species, measuring approximately 300 μm in body length, so more careful and precise observation is required and recommended for their examination.

Some specimens of *S. ivlevi* in the present study did not only show abnormal ornamentation items on the cephalosome or the genital double-somite (cf. Fig. 7B–E), but also featured morphological asymmetries and abnormalities on other body parts, such as the swimming legs. The atypical spine count on the right exp-3 of P2 observed in a female (robust form) from the Korea Strait showing two instead of the typical three spines (see above under “Remarks” of *S. ivlevi*) is of particular importance as the spine count of this leg is used for distinguishing *S. ivlevi* (three spines) from *S. humesi* (two spines). Therefore, identification of the two species simply based on leg armature may lead to misidentification and care should be taken to use additional morphological parameters for their distinction/differentiation. Morphological abnormalities in various appendages have also been reported for various other oncaeid species such as an aberrant number of spines on swimming leg 1 in Oncaea venusta f. typica Farran, 1929 (Böttger-Schnack 2001: fig. 4a); a modified tip of the posterolateral corner on P6 in males of *Triconia hawii* (Böttger-Schnack & Boxshall, 1990) (Böttger-Schnack 1999: fig. 23a–d); *Oncaea media* Giesbrecht, 1891 and *O. waldemari* Bersano & Boxshall, 1996 (Böttger-Schnack 2001: figs 16C, 27E) and *Triconia giesbrechti* Böttger-Schnack, 1999 (Cho et al. 2013: fig. 12F); an abnormal shape of the distal endopod segment of the antenna with an aberrant seta and a reduced number of setae (*Oncaea prolata* Heron, 1977 in Cho 2011: fig. 29I, male); an aberrant process on the outer proximal corner on the basis of P4 (*O. parabathyalis* Böttger-Schnack, 2005, in Böttger-Schnack 2005: fig. 18d, female); and the tumorous growth on the surface of the prosome of female *Triconia derivata* (Heron & Bradford-Grieve, 1995) (Heron and Bradford-Grieve 1995: figs 9h, 10a). Among marine pelagic copepods, the genus *Acartia* Dana, 1846 is a well-known taxon of morphological anomalies, mainly in P5. In a study on the morphological anomalies of *Acartia*, there was no indication found that the occurrence of anomalies on the P5 was relatively more frequent in the polluted area than in the open sea, and it was tentatively inferred that these anomalies may be a common phenomenon in nature (Brylinski 1984; Behrends et al. 1997). Also, morphological anomalies have been observed at the bases of P3 in *Clausocalanus mastigophorus* (Claus, 1863) collected from the equatorial Atlantic (Melo et al. 2014). It was assumed that this was due to a developmental error or random genetic mutations. Hence, in oncaeid copepods, the observed morphological asymmetries and abnormalities may be a common natural phenomenon as well, but further studies will be needed to provide sufficient information for avoiding any taxonomic confusion.

The present study included molecular genetic analyses with the aim of overcoming taxonomic problems related to morphological variation. The sequence of the mtCOI region could not successfully be obtained for any of the three species of *Spinoncaea* analyzed, supporting previous findings that for oncaeid copepods the 12S gene is a better tool for use in DNA barcoding than the COI gene (Böttger-Schnack and Machida 2011). The 12S srRNA sequences of *Spinoncaea* species from the Pacific did not indicate a genetic difference between species from the Mediterranean Sea and those from the northwestern and/or northeastern equatorial Pacific Ocean. However, morphological variation cannot exclude the possibility that these reflect population differences. And in the case of *Spinoncaea* species, especially *S. ivlevi*, the observed morphological variation from a broad geographical distribution may indicate a high level of gene flow between populations. Thus, mtDNA may not be an accurate indicator of species dispersal due to maternal inheritance of the organelle genome (Bucklin and Frost 2009). Nevertheless, genetic analysis of species with wide distributions and morphological variation in other copepod taxa has indicated the existence of pseudo-sibling species (Staton et al. 2005). In general, mtCOI sequence variation showed greater divergence between conspecific individuals collected in different regions or ocean basins (Bucklin et al. 2003). *Centropages typicus* Krøyer, 1849, showing clear morphological differences in the chela of the fifth thoracopod of the male, between the northwestern and northeastern Atlantic and the Mediterranean Sea showed genetic differences in mtCOI and nuclear rDNA ITS1 (Castellani et al. 2012). Recently, ITS rDNA (internal transcribed spacers of the nuclear ribosomal cistron) was used as a new marker in the molecular phylogeny of Oncaeidae, and this marker was also found to be useful for elucidating the genetic relationship between species (Di Capua et al. 2017). They proposed the use of ITS (especially ITS2) for phylogenetic reconstruction. Therefore, it can be suggested that our results will have to be discussed again in the future with further analysis of other regions of the gene (e.g., nuclear genes).

Recently, there was incongruence between the identified species of *Paracalanus parvus* complex through a comprehensive analysis of progressive molecular method and conventional morphology (Kasapidis et al. 2018). Therefore, considering the high morphological similarity of species belonging to the Oncaeidae, including *Spinoncaea*, the existence of sibling species, and the resulting complexity of taxonomic analyses, molecular analysis will be essential to clarify species identification in this taxon. To observe the relationships between morphological variation and genetic variation requires further analyses in the future taking into consideration various genes.

## Supplementary Material

XML Treatment for Spinoncaea ivlevi

XML Treatment for Spinoncaea tenuis

XML Treatment for Spinoncaea humesi
